# Reduced Density Matrix and Cumulant Approximations of Quantum Linear Response

**DOI:** 10.1021/acs.jctc.5c01353

**Published:** 2026-02-05

**Authors:** Theo Juncker von Buchwald, Erik Rosendahl Kjellgren, Jacob Kongsted, Stephan P. A. Sauer, Sonia Coriani, Karl Michael Ziems

**Affiliations:** † Department of Chemistry, 5205 Technical University of Denmark, Kemitorvet Building 207, Kongens Lyngby DK-2800, Denmark; ‡ Department of Physics, Chemistry and Pharmacy, 6174 University of Southern Denmark, Campusvej 55, Odense 5230, Denmark; § Department of Chemistry, 4321 University of Copenhagen, Copenhagen Ø DK-2100, Denmark; ∥ School of Chemistry, 7423 University of Southampton, Highfield, Southampton SO17 1BJ, United Kingdom

## Abstract

Linear response (LR) is an important tool in the computational chemist’s toolbox. It is therefore unsurprising that the emergence of quantum computers has led to a quantum counterpart known as quantum LR (qLR). However, the current quantum era of near-term intermediate-scale quantum (NISQ) computers is dominated by noise, short decoherence times, and slow measurement speeds. It is therefore of interest to find approximations that can greatly reduce the quantum workload while only slightly impacting the quality of a method. In an effort to achieve this, we approximate the naive qLR with the singles and doubles (qLRSD) method, by either directly approximating the reduced density matrices (RDMs) or indirectly through their respective reduced density cumulants (RDCs). We present an analysis of the measurement costs associated with qLR using RDMs and report qLR results for model hydrogen ladder systems; for varying active space sizes in OCS, SeH_2_, and H_2_S; and for symmetrically stretched H_2_O and BeH_2_. Discouragingly, while approximations to the 4-body RDMs and RDCs seem to produce good results for systems at the equilibrium geometry and for some types of core excitations, they both tend to fail when the system exhibits strong correlation. All approximations to the 3-body RDMs and/or RDCs severely affect the results and cannot be applied.

## Graphical abstract

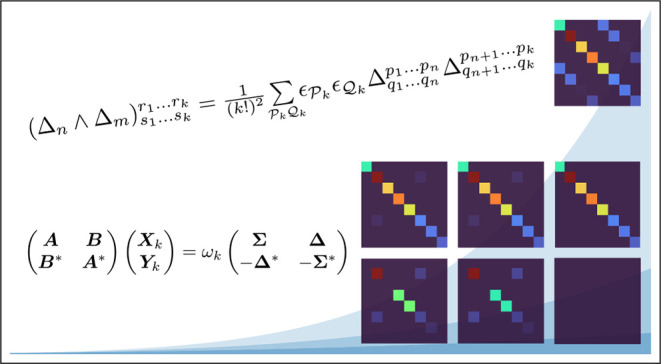

## 1 Introduction

Predicting molecular properties such as excitation energies, oscillator strengths, and rotational strengths plays a crucial role in interpreting spectroscopic data. This capability also serves as a foundation in various scientific fields, including photochemistry, photophysics, photocatalysis, and the design of photoactivatable products for novel therapeutic tools. To this end, the molecular response framework^1–4^ is an obvious choice to compute molecular properties, as it has long been established for conventional hardware and has, in the past years, been reformulated for quantum hardware.^5–8^

On conventional hardware, linear response (LR) has been formulated and implemented for a wide range of electronic structure methods, such as Hartree-Fock theory,^1,9^ multiconfigurational self-consistent field (MCSCF) theory,^1,10,11^ coupled cluster theory,^2,4,12^ Møller-Plesset perturbation theory,^13–15^ and time-dependent density functional theory.^16,17^

On the quantum side, linear response has been adapted to the quantum linear response (qLR) framework,^5,6,8,18–20^ the variational quantum response (VQR) method,^21^ and other general LR frameworks.^22,23^ Since its initial proposal,^5^ qLR has been further extended, e.g., with the development of an orbital-optimized variant for active spaces,^6^ a reduced density matrix implementation,^6,8^ a Davidson solver approach,^18^ and the introduction of polarizable embedding environments.^19^ Recently, multiple strategies to reduce the hardware requirements were utilized in a successful application of qLR on quantum hardware,^20^ along with a detailed analysis of the algorithm.^24,25^ Other approaches to calculate excited states and response properties using quantum hardware, such as quantum equation of motion,^7,26–28^ state-average VQE variants,^29–32^ and variational quantum deflation,^33,34^ have also been introduced.

Not only is the fault-tolerant evaluation of linear response functions currently out of reach, but even going beyond a few qubits is impossible on current quantum devices due to noise rates, decoherence, and sampling speed. Therefore, approximations to the qLR have been considered and carefully studied for their accuracy. In the classical regime, MCSCF is used to significantly reduce the computational requirements by only treating the correlation of the active space, thereby reducing the size of the problem. We note that MCSCF LR scales exponentially with the size of the active space. In the paper by Ziems et al.,^6^ an MCSCF approach to qLR was employed, where an active space was used to reduce the quantum hardware requirements. The active space approach was combined with orbital optimization and the truncation of excitation rank within the active space. In the same work, the authors introduced eight parametrization schemes, of which three were labeled as applicable in the near term. Of these, in a previous paper, we formulated the naive parametrization, restricted to singles and doubles excitation in the active space, in a reduced density matrix (RDM) formalism needing up to the four-body RDM (4-RDM) and reducing the classical aspects of the parametrization with the use of rank reduction.^8^ This allowed for the calculation of response properties for medium-sized molecules with moderately sized basis sets.

The RDM formalism, however, still comes at a relatively steep cost, since the 4-RDM scales with the number of orbitals in the active space to the eighth power, $\left(N_{A}^{8}\right)$ . It is therefore of interest to find approximations in an effort to further reduce the size of the problem. One possible approach would be to directly approximate the RDMs. Another approach would be to approximate the corresponding reduced density cumulants (RDCs)^35–37^ and reconstruct the RDMs. Approximations using RDCs are a well-known strategy within conventional quantum chemistry methods such as NEVPT2,^38^ CASPT2,^39^ DMRG,^40,41^ and DSRG.^42^ Inspired by these works, we here explore the possibility of using reduced density matrix and reduced density cumulant approximations in our formulation of naive quantum linear response to reduce the number of quantum measurements as well as the classical cost.

This work is organized as follows. In Section 2, we briefly review the active space approximation and the orbital-optimized unitary coupled cluster (oo-UCC) method, and then introduce the qLR framework. In Section 2, RDMs and RDCs, as well as their approximations, are introduced. In Section 3, we provide the computational details, followed by Section 4 where we start by providing a detailed analysis of the amount of quantum measurements depending on RDMs, active space, and mapping, as well as analyzing how this translates to the actual qLR algorithm. Next, we present results for our approximations, which are first applied to hydrogen ladder model systems of increasing size in a minimal basis, followed by various molecular systems in different active spaces. Lastly, we investigate the effects of strong correlation on the approximations. Concluding remarks are given at the end.

## 2 Theory

Throughout this paper, unless otherwise stated, the following notation will be used: *p*, *q*, *r*, *s*, *t*, *u*, *m*, and *n* for general orbital indices; *a, b, c,* and *d* for virtual (secondary) orbital indices; *i, j, k,* and *l* for inactive (doubly occupied) orbital indices; and ν_*a*_ and ν_*i*_ for active-space orbitals that are, respectively, virtual and doubly occupied in the Hartree-Fock reference state.

### 2.1 Active Space

In the active space approximation, the full space is divided into three subspaces: inactive, active, and virtual (secondary) space. The orbitals in the inactive space are considered to be doubly occupied; the orbitals in the virtual (secondary) space are empty, and the orbitals in the active space are then ordinarily treated with a full configuration interaction (FCI) expansion. With this approximation, only the active space part of an operator, *Ô*_A_, and the active space part of the wave function, |*A*(**θ**)⟩, are treated on the quantum computer.

The selection of the active space is commonly done by choosing orbitals and the number of electrons for the active-space part. This gives the notation (*N*_e_, *N*_A_), where *N*_e_ is the number of electrons included in the active space and *N*_A_ is the number of spatial orbitals in the active space. This strategy is famously used for the classical method CASSCF (complete active space self-consistent field),^43–45^ where the active space is treated using a complete CI expansion. Recently, the active space strategy has also been applied in quantum computing for chemistry, where only the active space is treated on the quantum device, whereas the inactive space and virtual space are treated classically.^46^ A popular parametrization of the active space is unitary coupled cluster (UCC), wherein a further approximation is the truncation of the cluster operator.^47–49^ The expectation value of an operator acting on the active space (i.e., being evaluated on quantum hardware) is treated by translating the operator to a sum of Pauli strings

$$\left\langle A\left(\theta \right)\left|{Ô}_{A}\right|A\left(\theta \right)\right\rangle =\underset{l}{\sum }c_{l}\left\langle A\left(\theta \right)\right|{\mathrm{P̂}}_{l}\left|A\left(\theta \right)\right\rangle$$ 1

Each Pauli string, *P̂*_*l*_, has a corresponding coefficient, *c*_l_, both of which are dependent on the mapping (for example, Jordan-Wigner,^50^ Parity,^51^ or Bravyi-Kitaev^52^) chosen. Different operators may share Pauli strings; therefore, the results of a given Pauli string measurement are saved in memory to be used for different operators.^20^ The measurement overhead can be further reduced by utilizing commuting groups,^53^ such as qubit-wise commutativity (QWC).^54^

### 2.2 Orbital-Optimized Unitary Coupled Cluster

An extension of UCC is the orbital-optimized unitary coupled cluster (oo-UCC),^47–49^ where UCC is performed in the active space, and the orbital optimization is performed between the inactive and active spaces, the active and virtual (secondary) spaces, and the inactive and virtual (secondary) spaces.

The UCC wave function is given as an exponential parametrization of a reference wave function |0⟩

2 $$\left|\mathrm{UCC}\left(\theta \right)\right\rangle =e^{\mathrm{T̂}(\theta )-{\mathrm{T̂}}^{†}\left(\theta \right)}\left|0\right\rangle$$

where *T̂*(**θ**) = *T̂*_1_(**θ**) + *T̂*_2_(**θ**) + ... is the cluster operator, which generates excited determinants, where

3 $${\mathrm{T̂}}_{1}\left(\theta \right)=\underset{\nu_{a}\nu_{i}}{\sum }\theta_{\nu_{a}\nu_{i}}{Ê}_{\nu_{a}\nu_{i}}$$

4 $${\mathrm{T̂}}_{2}\left(\theta \right)=\frac{1}{2}\underset{\begin{array}{l} \nu_{a}\nu_{i}\\ \nu_{b}\nu_{j} \end{array}}{\sum }\theta_{\nu_{a}\nu_{i}\nu_{b}\nu_{j}}{Ê}_{\nu_{a}\nu_{i}}{Ê}_{\nu_{b}\nu_{j}}$$

and *Ê*_*pq*_ is the singlet one-electron excitation operator, defined by the creation ${â}_{p\sigma }^{†}$ and annihilation *â*_*q*σ_ operators, ${Ê}_{pq}={â}_{p\alpha }^{†}{â}_{q\alpha }+{â}_{p\beta }^{†}{â}_{q\beta }$ . We used σ to denote an arbitrary spin of α or β. The adjoint cluster operator *T̂* ^†^(**θ**) generates all de-excitations and ensures unitarity. Like in CI and in regular CC, the cluster operator *T̂*(**θ**) (and *T̂* ^†^(**θ**)) can be truncated. As an example, UCC singles and doubles (UCCSD) only include the singles and doubles cluster operators. Unlike CI, the UCC method remains size-consistent when truncating the cluster operator.^55^

The orbital optimization is done by exponentially parametrizing the UCC wave function with the orbital rotation operator *κ̂*(**κ**)­

$$\left|\mathrm{oo‐UCC}\left(\kappa ,\theta \right)\right\rangle =e^{-\mathrm{\kappa ̂}(\kappa )}\left|\mathrm{UCC}\left(\theta \right)\right\rangle$$ 5

6 $$\mathrm{\kappa ̂}\left(\kappa \right)=\underset{p>q}{\sum }\kappa_{pq}{Ê}_{pq}^{-}$$

where ${Ê}_{pq}^{-}={Ê}_{pq}-{Ê}_{qp}$ only acts between the inactive, active, and virtual spaces. The **θ** and **κ** parameters are found by minimizing the energy using the orbital-optimized variational quantum eigensolver (oo-VQE)^48,49^

$$E\left(\kappa ,\theta \right)=\underset{\kappa ,\theta }{\min}\left\langle \mathrm{UCC}\left(\theta \right)\right|Ĥ\left(\kappa \right)\left|\mathrm{UCC}\left(\theta \right)\right\rangle$$ 7

where the Hamiltonian has been similarity transformed by the orbital rotation parameters.

### 2.3 Linear Response

As anticipated in Section 1, properties such as excitation energies, oscillator strengths, and rotational strengths can be calculated through the linear response framework. Specifically, for an MCSCF reference wave function, the excitation energies can be found by solving a generalized eigenvalue equation of the form^1,3^

8 $$E^{[2]}\beta_{k}=\omega_{k}S^{[2]}\beta_{k}$$

where **E** ^[2]^ is the electronic Hessian, **S** ^[2]^ is the metric, **β**_k_ is the excitation vector, and ω_*k*_ is the corresponding excitation energy for excited state *k*. The Hessian and metric matrices are defined in terms of the submatrices ***A***, ***B***, **Σ**, and **Δ**.

9 $$E^{[2]}=\left(\begin{array}{cc} A & B\\ B^{*} & A^{*} \end{array}\right),S^{[2]}=\left(\begin{array}{cc} \Sigma & \Delta \\ -\Delta^{*} & -\Sigma^{*} \end{array}\right)$$

The submatrices ***A*** and ***B*** involve double commutators between the Hamiltonian operator *Ĥ* and the orbital rotation operators *q̂*_*μ*_ and the active-space excitation operators *Ĝ*_*m*_,

10 $$A=\left(\begin{array}{cc} \left\langle 0\left|\left[{\mathrm{q̂}}_{\mu }^{†},\left[Ĥ,{\mathrm{q̂}}_{\nu }\right]\right]\right|0\right\rangle & \left\langle 0\left|\left[{\mathrm{q̂}}_{\mu }^{†},\left[Ĥ,{Ĝ}_{m}\right]\right]\right|0\right\rangle \\ \left\langle 0\left|\left[{Ĝ}_{n}^{†},\left[Ĥ,{\mathrm{q̂}}_{\nu }\right]\right]\right|0\right\rangle & \left\langle 0\left|\left[{Ĝ}_{n}^{†},\left[Ĥ,{Ĝ}_{m}\right]\right]\right|0\right\rangle \end{array}\right)$$

11 $$B=\left(\begin{array}{cc} \left\langle 0\left|\left[{\mathrm{q̂}}_{\mu }^{†},\left[Ĥ,{\mathrm{q̂}}_{\nu }^{†}\right]\right]\right|0\right\rangle & \left\langle 0\left|\left[{\mathrm{q̂}}_{\mu }^{†},\left[Ĥ,{Ĝ}_{m}^{†}\right]\right]\right|0\right\rangle \\ \left\langle 0\left|\left[{Ĝ}_{n}^{†},\left[Ĥ,{\mathrm{q̂}}_{\nu }^{†}\right]\right]\right|0\right\rangle & \left\langle 0\left|\left[{Ĝ}_{n}^{†},\left[Ĥ,{Ĝ}_{m}^{†}\right]\right]\right|0\right\rangle \end{array}\right)$$

and **Σ** and **Δ** involve commutators between the orbital rotation and the active-space excitation operators

12 $$\Sigma =\left(\begin{array}{cc} \left\langle 0\left|\left[{\mathrm{q̂}}_{\mu }^{†},{\mathrm{q̂}}_{\nu }\right]\right|0\right\rangle & \left\langle 0\left|\left[{\mathrm{q̂}}_{\mu }^{†},{Ĝ}_{m}\right]\right|0\right\rangle \\ \left\langle 0\left|\left[{Ĝ}_{n}^{†},{\mathrm{q̂}}_{\nu }\right]\right|0\right\rangle & \left\langle 0\left|\left[{Ĝ}_{n}^{†},{Ĝ}_{m}\right]\right|0\right\rangle \end{array}\right)$$

13 $$\Delta =\left(\begin{array}{cc} \left\langle 0\left|\left[{\mathrm{q̂}}_{\mu }^{†},{\mathrm{q̂}}_{\nu }^{†}\right]\right|0\right\rangle & \left\langle 0\left|\left[{\mathrm{q̂}}_{\mu }^{†},{Ĝ}_{m}^{†}\right]\right|0\right\rangle \\ \left\langle 0\left|\left[{Ĝ}_{n}^{†},{\mathrm{q̂}}_{\nu }^{†}\right]\right|0\right\rangle & \left\langle 0\left|\left[{Ĝ}_{n}^{†},{Ĝ}_{m}^{†}\right]\right|0\right\rangle \end{array}\right)$$

The excitation (column) vector obtained by solving eq 8 is in the form

14 $$\beta_{k}=\left(\begin{array}{c} Z_{k}\\ Y_{k}^{*} \end{array}\right)$$

where the excitation block ***Z***_*k*_ corresponds to ω_*k*_ > 0, and the de-excitation block $Y_{k}^{*}$ corresponds to ω_*k*_ < 0. Given the excitation vectors and the relevant property gradients, transition properties such as oscillator strengths can be calculated; see, e.g., refs 6 and 8.

In the paper by Ziems et al.,^6^ eight LR Ansätze were introduced through specific choices of the operators *q̂* and *Ĝ*. We focus here on the naive parametrization and truncate the linear-response excitation rank to spin-adapted singles and doubles excitations^56,57^ (which we will refer to as qLRSD from now on),

15 $${\mathrm{q̂}}_{pq}=\frac{1}{\sqrt{2}}{Ê}_{pq},\mathrm{with}\;pq=\left\{\nu i,ai,a\nu \right\}$$

and

16 $$\begin{array}{ll} Ĝ\in \{\frac{1}{\sqrt{2}}{Ê}_{v_{a}v_{i}}, & \frac{1}{2\sqrt{\left(1+\delta_{v_{a}v_{b}}\right)\left(1+\delta_{v_{i}v_{j}}\right)}}\left({Ê}_{v_{a}v_{i}}{Ê}_{v_{b}v_{j}}+{Ê}_{v_{a}v_{j}}{Ê}_{v_{b}v_{i}}\right),\\ & \frac{1}{2\sqrt{3}}\left({Ê}_{v_{a}v_{i}}{Ê}_{v_{b}v_{j}}-{Ê}_{v_{a}v_{j}}{Ê}_{v_{b}v_{i}}\right)\} \end{array}$$

Note that if the active space spans the full space, like in FCI, there is no inactive or virtual (secondary) space. Additionally, while in this paper we specifically chose to use an oo-UCC Ansatz, any quantum Ansatz for the active space works with the qLR algorithm.^24,25^

Equation 8 can be solved by explicitly constructing the elements of the submatrices entering the Hessian and metric, and then performing a diagonalization of the resolvent. In line with ref 8 we here adopt this strategy and investigate the accuracy of approximations made for the 3- and 4-RDM to reduce the quantum scaling of the qLR method.

### 2.4 Reduced Density Matrices

In our former study,^8^ we reformulated the construction of the Hessian and metric of naive qLRSD parametrization using reduced density matrices, which reduced the classical requirements by rank reduction, allowing larger basis sets to be employed. The RDM formulation affected the classical regime, allowing for more inactive and virtual orbitals. The Hessian, metric, and property gradient were then constructed using RDMs, where the active-space RDM contributions were evaluated on an (emulated) quantum device (see eqs S5–S28 in ref 8 for details on the working equations for the RDM-driven implementation of naive qLR).

When restricting the excitation rank in the active space in naive qLR to singles and doubles excitations, only the RDMs up to the 4-RDM are needed to construct the Hessian.^8^ In second quantization, the full 1-, 2-, 3-, and 4-RDM are given as

17 $$\begin{array}{lll} \Gamma_{pq}^{[1]} & = & \underset{\tau \in \left\{\alpha ,\beta \right\}}{\sum }\left\langle 0\left|{â}_{p\tau }^{†}{â}_{q\tau }\right|0\right\rangle \\ \Gamma_{pqrs}^{[2]} & = & \underset{\tau \gamma \in \left\{\alpha ,\beta \right\}}{\sum }\left\langle 0\left|{â}_{p\tau }^{†}{â}_{r\gamma }^{†}{â}_{s\gamma }{â}_{q\tau }\right|0\right\rangle \\ \Gamma_{pqrstu}^{[3]} & = & \underset{\tau \gamma \delta \in \left\{\alpha ,\beta \right\}}{\sum }\left\langle 0\left|{â}_{p\tau }^{†}{â}_{r\gamma }^{†}{â}_{t\delta }^{†}{â}_{u\delta }{â}_{s\gamma }{â}_{q\tau }\right|0\right\rangle \\ \Gamma_{pqrstumn}^{[4]} & = & \underset{\tau \gamma \delta \sigma \in \left\{\alpha ,\beta \right\}}{\sum }\left\langle 0\left|{â}_{p\tau }^{†}{â}_{r\gamma }^{†}{â}_{t\delta }^{†}{â}_{m\sigma }^{†}{â}_{n\sigma }{â}_{u\delta }{â}_{s\gamma }{â}_{q\tau }\right|0\right\rangle \end{array}$$

The computationally expensive step is measuring the 3- and 4-RDM in the active space, which scale as $O\left(N_{A}^{6}\right)$ and $O\left(N_{A}^{8}\right)$ , respectively. Therefore, it is favorable to find approximations for the RDMs. A straightforward way is to ignore (parts of) the 3- and 4-RDM. As summarized in Table 1, we investigate four direct approximations to the RDMs here, namely setting the complete (3- and) 4-RDM to zero or just their off-diagonals. We here refer to the “pairwise diagonal” as the diagonal: for a 4-dimensional tensor, *pqrs*, the diagonal would be *ppqq*.

**Table 1:** RDM and RDC Approximations

Approximation	Description
4-zRDM	Set the entire 4-RDM to zero
3-zRDM and 4-zRDM	Set the entire 3-RDM and 4-RDM to zero
4-dRDM	Set all off-diagonal elements of the 4-RDM to zero
3-dRDM and 4-dRDM	Set all off-diagonal elements of the 3-RDM and 4-RDM to zero
4-zRDC	Set the entire 4-RDC to zero
3-zRDC and 4-zRDC	Set the entire 3-RDC and 4-RDC to zero
4-dRDC	Set all off-diagonal elements of the 4-RDC to zero
3-dRDC and 4-dRDC	Set all off-diagonal elements of the 3-RDC and 4-RDC to zero

### 2.5 Reduced Density Cumulants

An alternative to introducing direct approximations in the RDMs is to reconstruct them from reduced density cumulants (RDCs).^35,37^ The 1-RDM through 4-RDM can be constructed by RDCs as

18 $$\Gamma^{[1]}=\Delta_{1}$$

19 $$\Gamma^{[2]}=\Delta_{1}\wedge \Delta_{1}+\Delta_{2}$$

20 $$\Gamma^{[3]}=\Delta_{1}\wedge \Delta_{1}\wedge \Delta_{1}+3\Delta_{2}\wedge \Delta_{1}+\Delta_{3}$$

21 $$\Gamma^{[4]}=\Delta_{1}\wedge \Delta_{1}\wedge \Delta_{1}\wedge \Delta_{1}+6\Delta_{2}\wedge \Delta_{1}\wedge \Delta_{1}+3\Delta_{2}\wedge \Delta_{2}+4\Delta_{3}\wedge \Delta_{1}+\Delta_{4}$$

where **Δ**_*n*_ is the *n*-RDC and **Δ**_*n*_ ∧ **Δ**_*m*_ is the Grassmann product of the *n*-RDC and *m*-RDC given by

22 $${\left(\Delta_{n}\wedge \Delta_{m}\right)}_{s_{1}...s_{k}}^{r_{1}...r_{k}}=\frac{1}{{\left(k!\right)}^{2}}\underset{P_{k}Q_{k}}{\sum }\epsilon_{P_{k}}\epsilon_{Q_{k}}\Delta_{q_{1}...q_{n}}^{p_{1}...p_{n}}\Delta_{q_{n+1}...q_{k}}^{p_{n+1}...p_{k}}$$

where P is the set of permutations over the upper indices, Q is the set of permutations over the lower indices, $P_{k}$ and $Q_{k}$ are elements of the sets of permutations, $\epsilon_{P_{k}}$ and $\epsilon_{Q_{k}}$ are the parities of permutations $P_{k}$ and $Q_{k}$ , and *k* = *n* + *m* is the total number of upper or lower indices. P and Q both contain *k*! permutations, and, as such, the summation runs over (*k*!)^2^ elements. The Grassmann product is commutative

23 A∧B=B∧A

associative

24 A∧(B∧C)=(A∧B)∧C

and antisymmetric in the upper and lower indices

25 $${\left(\Delta_{n}\wedge \Delta_{m}\right)}_{s_{1}...s_{\gamma }...s_{\delta }...s_{k}}^{r_{1}...r_{\mu }...r_{\nu }...r_{k}}=-{\left(\Delta_{n}\wedge \Delta_{m}\right)}_{s_{1}...s_{\gamma }...s_{\delta }...s_{k}}^{r_{1}...r_{\nu }...r_{\mu }...r_{k}}$$

26 $$=-{\left(\Delta_{n}\wedge \Delta_{m}\right)}_{s_{1}...s_{\delta }...s_{\gamma }...s_{k}}^{r_{1}...r_{\mu }...r_{\nu }...r_{k}}={\left(\Delta_{n}\wedge \Delta_{m}\right)}_{s_{1}...s_{\delta }...s_{\gamma }...s_{k}}^{r_{1}...r_{\nu }...r_{\mu }...r_{k}}$$

The expression for the *k*-RDC is found by isolation in the *k*-RDM expression in eqs 18–21

27 $$\Delta_{1}=\Gamma^{[1]}$$

28 $$\Delta_{2}=\Gamma^{[2]}-\Delta_{1}\wedge \Delta_{1}$$

29 $$\Delta_{3}=\Gamma^{[3]}-\Delta_{1}\wedge \Delta_{1}\wedge \Delta_{1}-3\Delta_{2}\wedge \Delta_{1}$$

30 $$\Delta_{4}=\Gamma^{[4]}-\Delta_{1}\wedge \Delta_{1}\wedge \Delta_{1}\wedge \Delta_{1}-6\Delta_{2}\wedge \Delta_{1}\wedge \Delta_{1}-3\Delta_{2}\wedge \Delta_{2}-4\Delta_{3}\wedge \Delta_{1}$$

Reconstructing the RDMs from the RDCs provides more possibilities for approximations, as summarized in Table 1. In the approximations called zRDC, the RDC of a given order is set to zero, and the RDM of the same order is partially reconstructed from the lower-order RDCs. For instance, in the 4-zRDC approximation, we use the exact 1-, 2-, and 3-RDMs and reconstruct the 4-RDM using eq 21 with **Δ**_4_ = 0, i.e., only utilizing the exact 1-, 2-, and 3-RDCs. In the dRDC approximations, the exact diagonal of the RDM is kept, and the off-diagonal elements of the RDM are partially reconstructed from the lower-order RDCs, as shown above. These approximations are employed for both 3- and 4-RDM, and for 4-RDM alone.

Since the summation in the Grassmann product runs over (*k*!)^2^ elements, the reconstruction of the *k*-RDM naively scales as $O\left\{N_{A}^{2k}\cdot {\left(k!\right)}^{2}\right\}$ , where $N_{A}^{2k}$ comes from the size of the *k*-RDM and (*k*!)^2^ comes from the summation in the Grassmann product. This scaling can be reduced to $O\left\{{\left(\frac{N_{A}!}{\left(N_{A}-k\right)!}\right)}^{2}\cdot \frac{1}{n!m!}\right\}$ by utilizing the antisymmetry of the Grassmann product and the symmetries of the RDCs:

- First, due to the antisymmetry of the Grassmann product, if two or more indices in the upper or lower indices are equal, the element must be zero. This reduces $N_{A}^{2k}$ to ${\left(\frac{N_{A}!}{\left(N_{A}-k\right)!}\right)}^{2}$ .
- Second, due to the antisymmetry of the Grassmann product, only elements that are unique in the upper and lower indices need to be calculated. Other elements may be found by permuting the indices to a known combination and multiplying with the parities of the permutations. As an example, take an element of the Grassmann product, ${\left(\Delta_{n}\wedge \Delta_{m}\right)}_{qsun}^{prtm}$ , such that *n* + *m* = 4, *p* > *r* > *t* > *m*, and *q* > *s* > *u* > *n*. From this element, all permutations of the indices *p*, *r*, *t*, and *m,* as well as all permutations of the indices *q*, *s*, *u*, and *n,* can be found. This reduces the scaling by a factor of (*k*!)^2^.
- Third, due to the symmetries of the RDCs, the number of permutations that need to be considered in the Grassmann product can be reduced. This reduces the scaling by a factor of *n*!*m*!.

One way to quantify the potential savings of the RDM and RDC approximations is the scaling of the methods—with a focus on the quantum workload, meaning the construction of the RDMs. With no approximations, the quantum workload scales as $O\left(N_{A}^{8}\right)$ due to the 4-RDM. The 4-zRDM and 4-zRDC approximations all scale as $O\left(N_{A}^{6}\right)$ due to the scaling of the 3-RDM. The 4-dRDM and 4-dRDC approximations also scale as $O\left(N_{A}^{6}\right)$ since the diagonal of the 4-RDM is in the computational basis and thus requires no additional measurements. Based on this, it is evident that the 3-RDM and 3-RDC approximations all scale as $O\left(N_{A}^{4}\right)$ in the number of measurements due to the 2-RDM. The scaling investigation can be extended to the classical regime through the construction of the Hessian. The construction of the Hessian is dominated by the active–active sub-blocks, which scale as $O\left(N_{A_{\mathrm{occ}}}^{4}N_{A_{\mathrm{unocc}}}^{4}N_{I+A}^{2}\right)$ , where $\left(N_{A_{\mathrm{occ}/\mathrm{unocc}}}\right)$ is the number of orbitals in the active space that are occupied/unoccupied in the Hartree-Fock reference, and (*N*_*I*+*A*_) is the number of inactive and active indices. The 4-RDM and 4-RDC approximations scale as $O\left(N_{A_{\mathrm{occ}}}^{3}N_{A_{\mathrm{unocc}}}^{3}N_{I+A}^{2}\right)$ and the 3-RDM and 3-RDC approximations scale as $O\left(N_{A_{\mathrm{occ}}}^{2}N_{A_{\mathrm{unocc}}}^{2}N_{I+A}^{2}\right)$ . For non-naive qLR methods discussed in ref 6 and for the quantum subspace expansion^58^ (QSE), these all scale as $O\left(N_{A}^{12}\right)$ . Thereby, the RDM- and RDC-based approximations could greatly reduce the quantum workload compared with other methods.

## 3 Computational Details

First, we quantify the potential savings of our approximations in terms of the number of measurements on a quantum device by calculating the number of Pauli strings needed for each approximation. This is done, for increasing active-space sizes, for both the Jordan-Wigner, Parity,^51^ and the Bravyi-Kitaev^52^ mappings. Furthermore, we utilize QWC to reduce the number of Pauli strings. To this end, the Pauli strings needed for a given RDM are simply sorted in reverse alphanumerical order and then grouped using QWC and first-fit bin packing. Note that the computational-basis string is always included in the list.

For the analysis of the eight approximations (Table 1) to naive qLRSD in the RDM framework, we investigate their effects on the increasing sizes of active spaces. For this purpose, we use ladders of dihydrogen molecules of various lengths in the minimal basis STO-3G,^59–61^ with a FCI wave function as our ansatz. The FCI ansatz parameters and the RDMs were obtained using PySCF.^62,63^ The H–H bond distance in the dihydrogen molecules (the rung) is 2.0 Bohr, and the distance between the dihydrogens (the distance between the rungs) is 1.5 Bohr. The qLR calculation is also performed in the full space (all electrons in all orbitals) with the excitation rank reduced to singles and doubles excitations (qLRSD) using our in-house DensityMatrixDrivenModule (DMDM).^64^ The integrals needed in DMDM are gathered through the PySCF interface to LIBCINT.^65^

Going beyond model systems, we also investigate the effects of the RDM and RDC approximations on chemically stable molecules with active spaces. For this purpose, we use the test systems shown in Table 2, where the number of doubly occupied (inactive) and virtual orbitals in the Hartree-Fock reference is given, along with the utilized active space. All calculations adopted the STO-3G basis set, and all wave function optimizations were performed at the oo-UCCSD level using SlowQuant,^66^ after which a qLRSD calculation was performed using DMDM.

**Table 2:** List of Molecules Considered, with the Number of Inactive (Doubly Occupied), $N_{I}^{\mathrm{HF}}$ , and Virtual, $N_{V}^{\mathrm{HF}}$ , Orbitals in the Hartree-Fock Reference State, and the Active Spaces Used^a^

Molecules	$N_{I}^{\mathrm{HF}}$	$N_{V}^{\mathrm{HF}}$	Active spaces
H_2_S	9	2	(4, 4); (8, 6)
OCS	15	4	(4, 4); (6, 6)
SeH_2_	18	2	(4, 4); (8, 6)

a The notation (*N*_*e*_, *N*_*A*_) is used for the active space, where *N*_*e*_ is the number of active electrons and *N*_*A*_ is the number of active orbitals .

To test the RDM/RDC approximations for strongly correlated systems, we additionally symmetrically stretch the geometry of H_2_O and BeH_2_ to 1.0, 1.5, and 2.0 times the equilibrium bond lengths of 0.92 and 1.35 Å, respectively. This is done using the cc-pVDZ^67^ basis set in the (6, 6) active space for water, and the cc-pVDZ basis set in both a (4, 4) and a (6, 6) active space for BeH_2_. Also, in these cases, calculations are performed with an oo-UCC ansatz using SlowQuant, after which a qLRSD calculation is performed on top using DMDM.

## 4 Results and Discussion

### 4.1 Pauli Savings

Table 3 collects the number of unique Pauli strings that are needed to measure the RDMs when using the Jordan-Wigner, Parity, and Bravyi-Kitaev transformations. Note that the number of Pauli strings for the RDMs is a system-independent variable—that is, it remains the same for different molecules when the same size of active space is used. The system-specific information is encapsulated in the state vector (Ansatz) that the RDM operators act on, as well as the integrals.

**Table 3:** Number of Additional Pauli Strings to Be Measured for Each Order of RDMs in Various Active Space Sizes using Jordan-Wigner, Bravyi-Kitaev, and Parity Mapping^a^

Active space	1-RDM	2-RDM	3-RDM	4-RDM
Jordan-Wigner mapping				
(4, 4)	17	273	834	785
(4, 6)	37	1,403	10,838	37,106
(6, 6)	37	1,403	10,838	37,106
(8, 6)	37	1,403	10,838	37,106
(10, 6)	37	1,403	10,838	37,106
(8, 8)	65	4,498	67,037	446,726
Parity mapping				
(4, 4)	15	234	336	144
(4, 6)	35	1,369	9,320	24,787
(6, 6)	35	1,369	9,325	25,099
(8, 6)	35	1,369	9,320	24,787
(10, 6)	35	1,369	9,325	25,099
(8, 8)	63	4,524	62,258	387,835
Bravyi-Kitaev mapping				
(4, 4)	15	231	339	144
(4, 6)	59	1,931	10,473	37,375
(6, 6)	59	1,931	10,473	37,375
(8, 6)	59	1,931	10,473	37,375
(10, 6)	59	1,931	10,473	37,375
(8, 8)	67	4,827	53,990	297,466

a For the QWC reduction, the Pauli strings are ordered reverse alphanumerically, with the computational-basis string always included. The notation (*N*_*e*_, *N*_*A*_) is used for the active space, where *N*_*e*_ is the number of active electrons and *N*_*A*_ is the number of active orbitals.

Each entry in the table corresponds to the additional number of Pauli strings needed to measure the *k*-RDM when compared to the (*k* – 1)-RDM. The trivial trend of an increasing number of Pauli strings with increasing active space size is observed for all mappings. In the Jordan-Wigner and Bravyi-Kitaev mappings, the number of electrons in the active space has no effect on the number of Pauli strings, so the mapping is dependent only on the number of orbitals in the active space. The Parity mapping shows a small dependency on the number of electrons in the active space, with an oscillatory behavior between even and odd numbers of α/β electrons. For the active spaces considered here, the Jordan-Wigner mapping consistently requires more Pauli-string measurements than the Parity and Bravyi-Kitaev mappings. The Parity mapping initially requires fewer Pauli string measurements than the Bravyi-Kitaev mapping, but this changes for the (8, 8) active space, where Bravyi-Kitaev requires the fewest Pauli string measurements.

It is apparent from Table 3 that a substantial amount of measurements can be saved by approximating the 3- and 4-RDMs, with greater savings at larger active spaces, thus motivating our approximations (Table 1). The *k*-zRDM and *k*-zRDC approximations require zero additional measurements compared to the (*k* – 1)-RDM, as both approximations are equivalent to not measuring the *k*-RDM. As the diagonals of the RDMs are in the computational basis, which is always included, no additional Pauli strings need to be measured for the 3-d and 4-dRDMs either. Due to this, the amount of measurements needed for each of the 3- and 4-RDM approximations is equivalent, and the primary difference between them comes down to classical costs.

Having seen that the 4-RDM is by far the largest contributor to the measurement costs, we next want to understand how many unique elements of the 4-RDM are used in the oo-naive qLR algorithm. Importantly, this is independent of the mapping used and depends on only the number of electrons and orbitals in the active space. The orbital optimization does not use elements of the 4-RDM, so this discussion is equally valid for naive qLR and oo-naive qLR.

In Table 4, we show that **(I)** as the number of orbitals in the active space increases, so does the percentage of the 4-RDM that is used, and **(II)** for a fixed number of orbitals, the number of symmetry-unique elements from the 4-RDM that are accessed in the construction of the Hessian in a given active space has a maximum when there are as many electrons as orbitals in the active space. Both of these trends stem from the number of *Ĝ* operators. This is due to an increase in allowed index combinations of the Hartree-Fock reference. To this end, trend **(II)** is equivalent to maximizing the function $\frac{V^{2}\cdot O^{2}}{{\left(V+O\right)}^{4}}$ , where *O* and *V* are the numbers of orbitals in the active space that are occupied and virtual in the Hartree-Fock reference, respectively. For a given active space, *V* + *O* is constant. The maximum of this function is found at *V* = *O*, i.e., an active space with an equal number of electrons and orbitals. This indicates that for strongly correlated systems, where the 4-RDM is more populated, the 4-RDM and 4-RDC approximations may produce significant errors.

**Table 4:** Number of Symmetry-Unique Elements Accessed from the 4-RDM in the oo-Naive qLR Algorithm During the Construction of the Hessian in a Given Active Space

Active space	Elements accessed	Total elements	Percent accessed
(4, 4)	1,761	1,996	88.23%
(4, 6)	35,800	41,406	86.46%
(6, 6)	37,415	41,406	90.36%
(8, 6)	35,311	41,406	85.28%
(10, 6)	26,785	41,406	64.69%
(8, 8)	349,877	384,112	91.09%
(10, 10)	2,022,907	2,212,750	91.42%
(12, 12)	8,555,818	9,340,332	91.60%

### 4.2 H_2_ Ladders

We start by assessing the impact of our eight approximations on the excitation energies and absorption spectra of H_2_ ladders of various lengths. The approximations to the RDMs and RDCs have the possibility of introducing linear dependencies in the linear-response equations. This leads to nonphysical excitation energies with a value of 0 Ha. In our investigations we remove these and refer to the remainder as ″non-zero excitation energies″. In Figure 1, the absorption spectra of 3 H_2_ and 6 H_2_ are shown (1 H_2_, 2 H_2_, 4 H_2_, and 5 H_2_ in Figure S1), while Table 5 quantifies the mean absolute errors (MAE) compared to FCI for a maximum of 100 non-zero excitation energies (the oscillator strengths can be found in Table S1). Table S2 quantifies the MAE compared to the FCI for all bright states under 30 eV. A state is here defined as bright when it has an oscillator strength above 0.01.

**Table 5:** Mean Absolute Errors (MAE) in eV for the Eight RDM and RDC Approximations for a Maximum of 100 Non-Zero Excitation Energies for Different Lengths of the H_2_ Ladder

	RDM approximations				RDC approximations			
System	4-z	3- and 4-z	4-d	3- and 4-d	4-z	3- and 4-z	4-d	3- and 4-d
1 H_2_	0.0000	0.0000	0.0000	0.0000	0.0000	0.0000	0.0000	0.0000
2 H_2_	0.2999	4.2836	0.2999	2.2334	0.2999	4.2835	0.2999	2.2291
3 H_2_	0.4273	7.4679	0.4273	1.6097	0.4414	7.0933	0.4296	1.7333
4 H_2_	0.8669	9.6414	0.8669	4.9432	0.8443	8.2002	0.8443	2.0725
5 H_2_	0.6302	12.4120	0.6302	4.8132	0.6972	10.4634	0.6972	2.7017
6 H_2_	0.8699	15.5930	0.8699	8.0726	0.8781	14.0385	0.8781	3.1770

**Figure 1:**
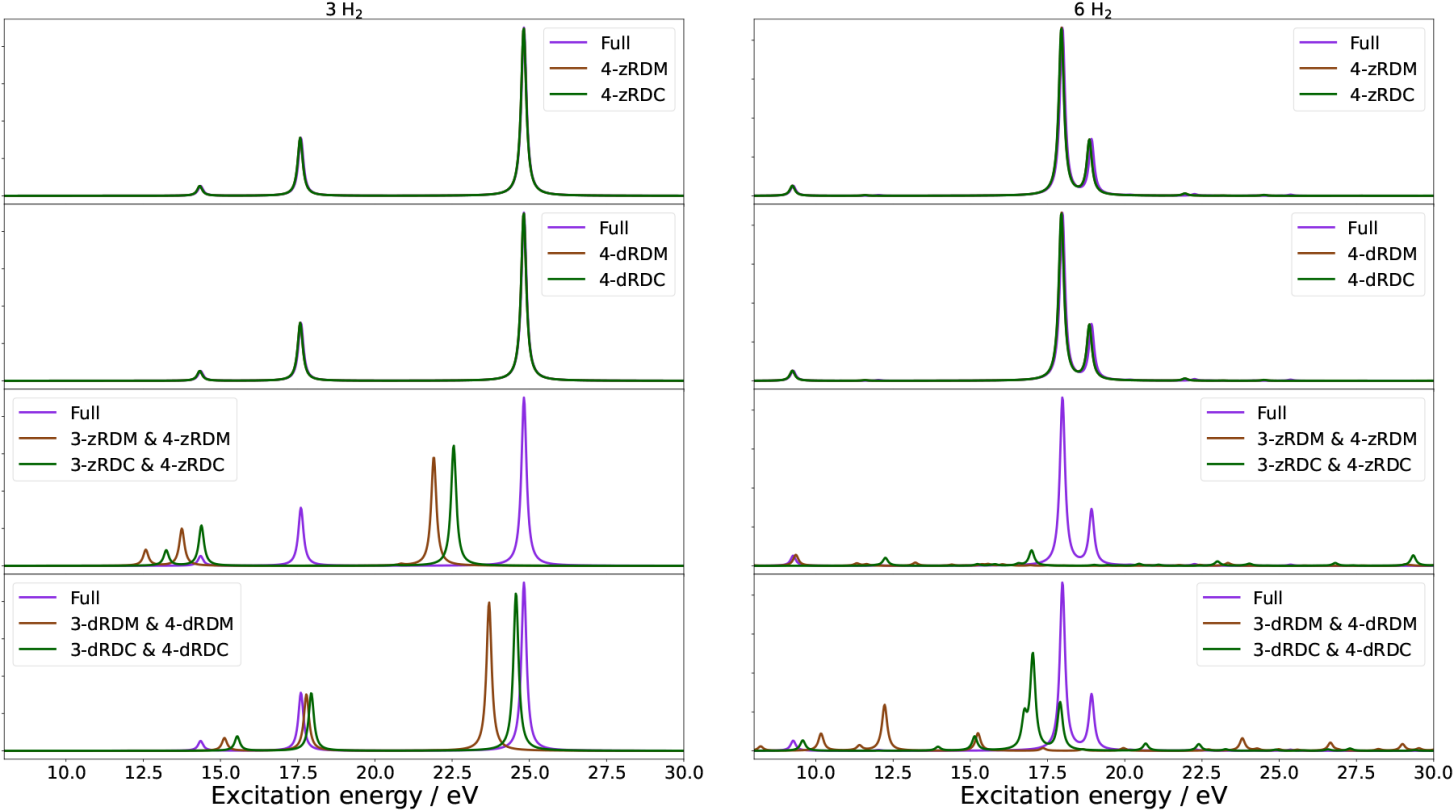
Absorption spectra of H_2_ ladders containing three (left) and six (right) rungs. Each figure contains four panels comparing the naive qLRSD absorption spectrum with no approximation to the absorption spectrum of naive qLRSD using the 4-zRDM and 4-zRDC approximations (first panel), the absorption spectrum of naive qLRSD using the 4-dRDM and 4-dRDC approximations (second panel), the absorption spectrum of naive qLRSD using the 3-zRDM and 4-zRDM and 3-zRDC and 4-zRDC approximations (third panel), and the absorption spectrum of naive qLRSD using the 3-dRDM and 4-dRDM and 3-dRDC and 4-dRDC approximations (fourth panel).

It is immediately clear from Figure 1 that approximations to the 4-RDM, whether done by approximating/neglecting the 4-RDM itself or its cumulant, have little qualitative impact on the spectra of the qLRSD method, while increasingly large errors are seen in Tables 5 and S2 with an increasing number of H_2_. From the MAE in Table S2, it is also clear that there is no difference between neglecting the entire 4-RDM (4-zRDM) or neglecting only its off-diagonal values (4-dRDM). This is further supported by counting the number of times that diagonal values of the 4-RDM are accessed, where, for all lengths of the H_2_ ladder, the diagonal 4-RDM values are accessed zero times. It is also apparent from Table S2 that approximations to the 4-RDC perform similarly to direct approximations of the 4-RDM. From the results in Tables 5 and S2, a general trend of the MAE rising with an increase in system size can be seen, in line with the trend seen in Table 4 of an increase in RDM usage with system size.

When we look at approximations to the 3-RDM, we can see in the bottom two panels of Figure 1 that any approximations to the 3-RDM, whether by approximating the RDM itself or its cumulant, significantly impact the spectrum. Therein, the 3- and 4-dRDM approximations perform better than the 3- and 4-zRDM approximations, and both 3-RDC approximations perform better than the respective 3-RDM approximations. The latter effect is more pronounced in the 3- and 4-dRDC approximation and, therefore, shows that the diagonal of the 3-RDC contributes appreciably to the excitation energies.

A comparison of Tables 5 and S2 shows how the majority of the error originates from dark states. This can also be seen in Figure 1, where the 4-RDM approximations do not qualitatively change the spectrum, yet still have MAEs of around 0.4 to around 0.8 eV, as seen in Table 5. However, when only comparing bright states, these errors fall to around 0.02 and 0.04 eV, with an outlier of 0.3 from 4 H_2_. This is caused by the bright excitations being dominated by single excitations, which do not require the 4-RDM.

To provide further detail on why the 3-RDM and 3-RDC approximations fail, we investigate the eigenvalue spectrum of the Hessian and metric of 4 H_2_. The eigenvalue ranges can be found in Table S3. Note that changes to the 4-RDM do not impact the metric, as is also evident from eqs 28 and 29 in ref 8. It is clear that any approximation of the 3-RDM or 3-RDC leads to negative eigenvalues in the Hessian while significantly shifting the spectrum—the shift being more pronounced for the zero approximations than for the diagonal approximations. Unfortunately, it cannot be guaranteed that only uninteresting excitations are affected by this change in the eigenvalue spectrum. It should be noted that, for the 3- and 4-zRDM approximation, there are eigenvalues in the Hessian and metric that change by a factor of 27.7 and 3.75, respectively.

A shot noise investigation of 2 H_2_ can be found in theSI. Here, we show that for 2 H_2_ the size of the errors caused by shot noise does not overshadow the errors caused by the RDM approximations themselves. These shot noise errors are expected to increase with the size of the active space.

### 4.3 Other Molecules

Next, we investigate whether the findings of the H_2_ model ladders hold true for a set of more realistic molecules. Special interest is in validating (or disproving) the previous finding that the 4-RDM can be neglected/approximated for the qLR algorithm.

In Figure 2, the absorption spectra of the molecules H_2_S, OCS, and SeH_2_ in the active spaces of (8, 6), (6, 6), and (8, 6) are shown; Table 6 collects the MAE of the first 100 non-zero excitation energies of H_2_S, OCS, and SeH_2_ in all the calculated active spaces. Results for the (4, 4) active space calculations, in addition to the 3-RDM and 3-RDC approximations, may be found in the SI in Figures S2 and S3. For standard deviations and values regarding oscillator strengths, we refer to Table S4. The green and brown curves overlap in the majority of the spectra and only deviate slightly in a few places.

**Table 6:** Mean Absolute Errors (MAE) in eV for the Eight RDM and RDC Approximations for a Maximum of 100 Non-Zero Excitation Energies^a^

	RDM approximations				RDC approximations			
Molecule	4-z	3- and 4-z	4-d	3- and 4-d	4-z	3- and 4-z	4-d	3- and 4-d
H_2_S (4, 4)	0.0748	1.0614	0.0748	0.6222	0.0748	1.0614	0.0748	0.6227
H_2_S (8, 6)	0.3286	1.4537	0.3286	1.1384	0.3295	1.3105	0.3295	1.1497
OCS (4, 4)	0.0835	26.5346	0.0835	25.3310	0.0835	26.5340	0.0835	25.3311
OCS (6, 6)	0.5964	10.1088	0.5964	5.9769	0.5934	13.3942	0.5934	3.8050
SeH_2_ (4, 4)	0.0368	0.4996	0.0368	0.3318	0.0368	0.4995	0.0368	0.3318
SeH_2_ (8, 6)	0.2327	1.1153	0.2327	0.6882	0.2341	1.0362	0.2341	0.8265

a The notation (*N*_*e*_, *N*_*A*_) is used for the active space, where *N*_*e*_ is the number of active electrons and *N*_*A*_ is the number of active orbitals .

**Figure 2:**
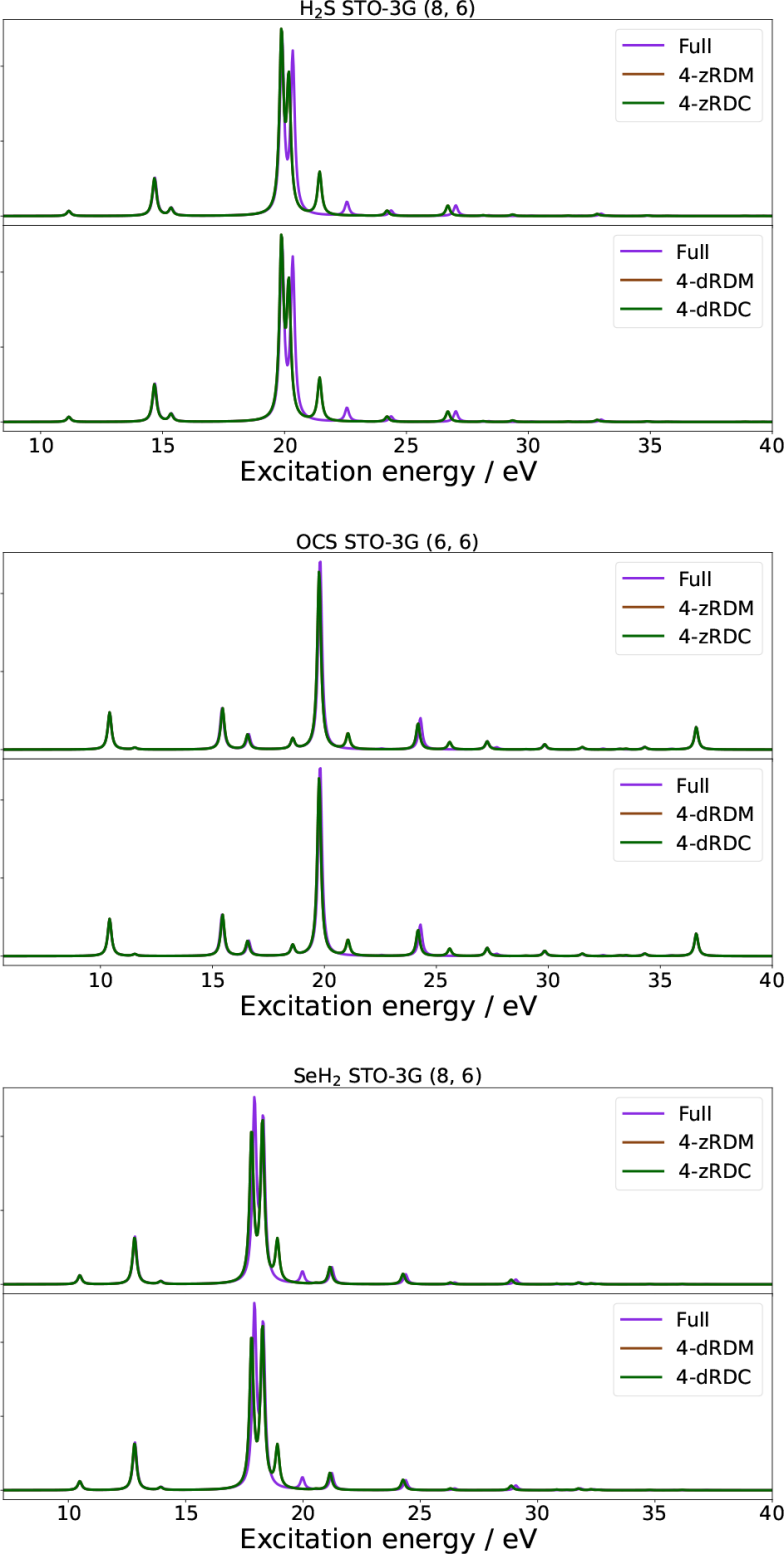
Absorption spectra of H_2_S (8, 6) [top], OCS (6, 6) [middle], and SeH_2_ [bottom]. Each figure contains two panels comparing the naive qLRSD absorption spectrum with no approximation to the absorption spectrum of naive qLRSD using the 4-zRDM and 4-zRDC approximations (first panel), and the absorption spectrum of naive qLRSD using the 4-dRDM and 4-dRDC approximations (second panel).

The results in Table 6 confirm that, in general, the contribution from the 4-RDM is very small, with a maximum MAE of 0.5964 ± 0.6293 eV for the excitation energies of OCS in a (6, 6) active space in the 4-zRDM approximation. While the trend of increasing errors with an increasing active space is apparent, the errors are smaller due to the contribution from the classically treated inactive space. This reduces the overall contribution from the active space to the excitation energies and thereby the errors caused by the approximations. Once again, the errors increase when approximating the 3-RDM to such a degree that it is not applicable to approximate the 3-RDM or its cumulant. It is noted that the 3- and 4-dRDC approximations are the best-performing variant of the 3-RDM approximations.

### 4.4 Strongly Correlated Systems

We have seen that, for molecules in their equilibrium geometry, the 4-RDM does not contribute significantly to the qLR algorithm. Since the 4-RDM is important for the qLR Hessian element with double excitations, the lack of relevance of the 4-RDM could be due to a dominance of single excitations at the equilibrium geometry. In order to further test our approximations, we turn our attention to more strongly correlated systems. Thus, in Figures 3 and 4, we show the valence absorption spectra of H_2_O and BeH_2_ for symmetric stretches of, respectively, the O–H and Be–H bonds to 1.0, 1.5, and 2.0 times the equilibrium bond lengths (*R*_eq_) of 0.92 Å and 1.35 Å. For both systems, we used a (6, 6) active space and the cc-pVDZ basis set.

**Figure 3:**
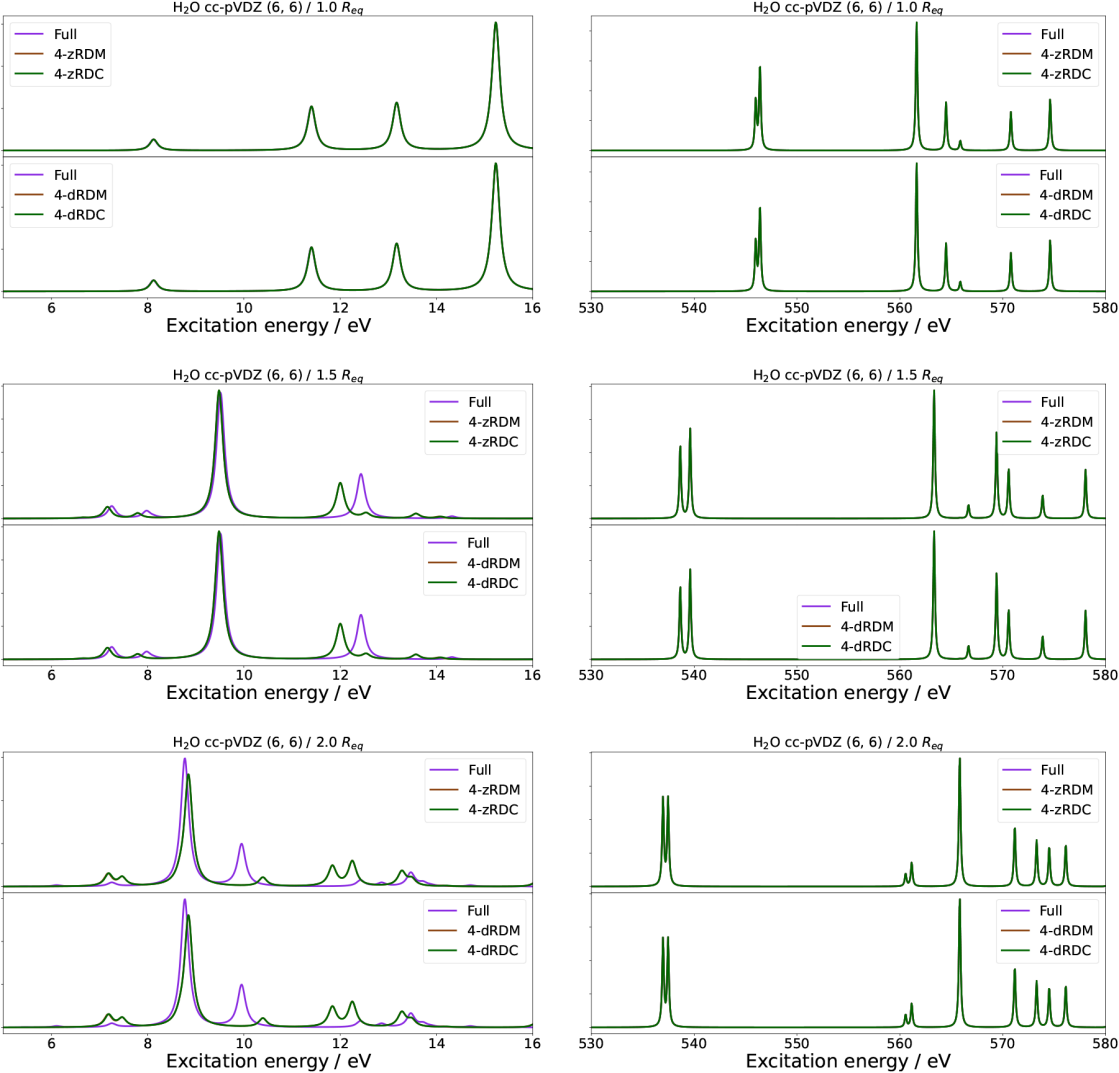
Absorption spectra of H_2_O in a (6, 6) active space with the cc-pVDZ basis set at differing symmetric stretches of the O–H atoms in the valence (left) and core (right) excitation regions. Each figure contains two panels comparing the naive qLRSD absorption spectrum with no approximation to the absorption spectrum of naive qLRSD using the 4-zRDM and 4-zRDC approximations (first panel), and the absorption spectrum of naive qLRSD using the 4-dRDM and 4-dRDC approximations (second panel).

**Figure 4:**
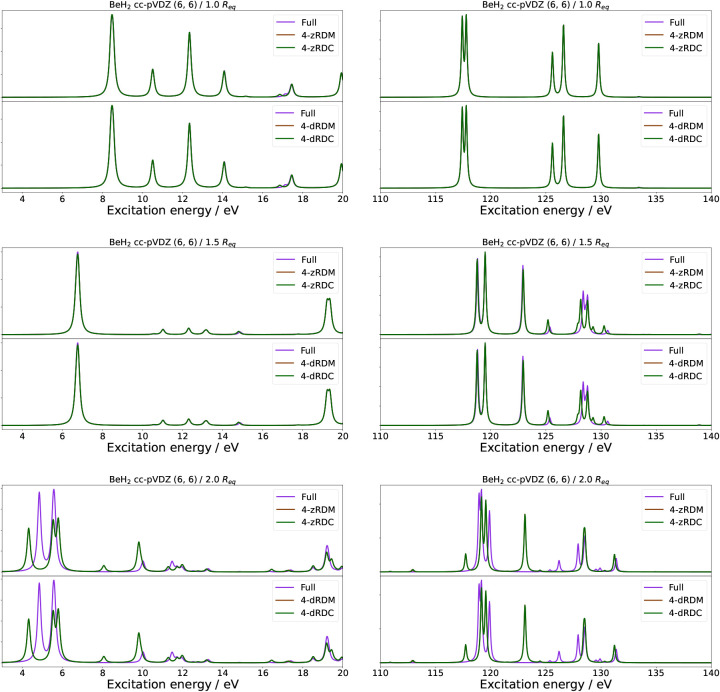
Absorption spectra of BeH_2_ in a (6, 6) active space with the cc-pVDZ basis set at differing symmetric Be–H stretches in the valence (left) and core (right) excitation regions. Each figure contains two panels comparing the naive qLRSD absorption spectrum with no approximation to the absorption spectrum of naive qLRSD using the 4-zRDM and 4-zRDC approximations (first panel), and the absorption spectrum of naive qLRSD using the 4-dRDM and 4-dRDC approximations (second panel).

We will here refer to valence excitation energies and core (K-edge) excitation energies for H_2_O and BeH_2_. Specifically, we define the valence excitation energies to be between 5 and 16 eV for H_2_O and between 3 and 20 eV for BeH_2_. For the core excitation energies, we define them to be between 530 and 580 eV for H_2_O and between 110 and 140 eV for BeH_2_. Further examples can be found in the SI, namely for the K-edge of oxygen and beryllium with different active space combinations, as well as results for the 3-RDM and 3-RDC approximations (see Figures S5 – S9 and Tables S5 – S8).

We continue to see the trend that RDC approximations perform on par with the RDM approximations at the equilibrium structure. At the same time, the 4-z and 4-d approximations perform the same within the RDM and RDC approximations, while the 3- and 4- approximations continue to have large errors for the valence excitation energies. With an elongation of the bond, the error increases and becomes significant even for the 4-RDM approximations. As expected, this is due to the strongly correlated systems’ reliance on double excitation contributions expressed by the 4-RDMs.

The core excitation energies paint a different picture. For H_2_O in a (6, 6) active space, the errors are very small for all bond lengths. This can be attributed to the core excitation energies being in the inactive space and thus described by single excitations that are, in turn, dominated by lower-order RDMs. In the case of H_2_O, this would allow for the core excitation energies to be calculated without the 3- and 4-RDM (as long as they are single excitation dominated), as seen in Figure S5. A similar trend can be observed for BeH_2_(4, 4) (see Figure S7). However, if the core electrons are placed in the active space, as done for BeH_2_ (6, 6) in Figure S9, this is no longer the case, and the errors of the core excitation energies at large bond distances become comparable to the errors of the valence excitation energies.

## 5 Summary

We have investigated eight approximations to our previously derived reduced density matrix formulation of naive orbital-optimized quantum linear response^8^ in order to reduce both the classical and quantum computational demands. The approximations are differentiated by first being applied to either only the 4-RDM or both the 3- and 4-RDM, and second by (I) keeping only the diagonal of an RDM, (II) discarding the entire RDM, (III) reconstructing the entire RDM from exact lower-order RDCs, and (IV) keeping the exact diagonal elements of the RDM and reconstructing the off-diagonal elements from exact lower-order RDCs.

We start by highlighting the measurement costs of evaluating the RDMs explicitly in various mapping schemes. As the 4-RDM scales $N_{A}^{8}$ , it clearly dominates the measurement cost, and approximations to it can drastically reduce the computational workload. For example, for a system with an (8, 8) active space, the 4-RDM cost dominates the overall costs by more than a factor of 5.

Applying our approximations to the excitation energies and absorption spectra of the H_2_ ladder model system, we concluded that entirely removing the 4-RDM only resulted in slight errors, while any approximation to the 3-RDM resulted in huge errors. When including the diagonal of the 4-RDM, no changes were observed; however, including the diagonal of the 3-RDM improved the performance compared to completely removing the entire 3-RDM. All errors increased with the size of the active space. Moving to chemically stable molecules confirmed the trends of the model system conclusion. In fact, the 4-RDM approximations had slightly smaller errors than in the H_2_ ladders, and the 3-RDM approximations continued to give huge errors, making them unusable. We still note that the diagonal 3-RDC approximations perform the best out of all 3-RDM and 3-RDC approximations.

For strongly correlated systems, here studied via stretched H_2_O and BeH_2_, we show that the 4-RDM cannot be ignored for high-quality results, as double excitations become important. Only core-excitation energies with the core orbitals in the inactive space (i.e., single excitations) are insensitive to our approximations regardless of bond length. In fact, in these spectra, there were little to no errors caused by disregarding the 3-RDM as well. However, when the core orbitals are included in the active space, as in BeH_2_ (6, 6), any approximations to the RDMs lead to large errors in the presence of strong correlation.

In summary, we showed that qLRSD can produce good results using approximations to the 4-RDM or 4-RDC for equilibrium systems or core excitations but struggles when either approximations to the 3-RDM or 3-RDC are introduced, or when the system exhibits strong correlation, which limits the potential applicability to quantum computing.

## Supplementary Material



## Data Availability

The data that support the findings of this study are available in the indicated SI and from the corresponding authors upon reasonable request.

## Notes

Additional tables and figures: mean absolute errors (MAE) and standard deviations (σ) for the eight RDM approximations in the H_2_ ladders; absorption spectra of H_2_ ladders containing one, two, four, and five links; mean absolute errors (MAE) for the eight RDM and RDC approximations for all bright states (osc. str. > 0.01) below 30 eV for different lengths of the H_2_ ladder; minimum and maximum eigenvalues of the Hessian and metric of 4 H_2_; MAE, σ, and absorption spectra for other molecules; MAE, σ, and absorption spectra (both valence and core) of strongly correlated systems; shot noise investigation for the 2H_2_ system (PDF)
