# Supplementary material for: Reduced Density Matrix and Cumulant Approximations of Quantum Linear Response
Source: J Chem Theory Comput. 2026 Feb 5;22(4):1652–63. doi: 10.1021/acs.jctc.5c01353 (PMC12937105; doi:10.1021/acs.jctc.5c01353)
Supplement: Supplementary file 1 [file ct5c01353_si_001.pdf]

Supporting Information: Reduced Density Matrix and Cumulant Approximations of Quantum Linear Response

Theo Juncker von Buchwald,[*,†] Erik Rosendahl Kjellgren,[*,‡] Jacob Kongsted,[‡] Stephan P. A. Sauer,[¶] Sonia Coriani,[†] and Karl Michael Ziems[*,§]

†*Department of Chemistry, Technical University of Denmark, Kemitorvet Building 207, DK-2800 Kongens Lyngby, Denmark.*

‡*Department of Physics, Chemistry and Pharmacy, University of Southern Denmark, Campusvej 55, 5230 Odense, Denmark.*

¶*Department of Chemistry, University of Copenhagen, DK-2100 Copenhagen Ø.*

§*School of Chemistry, University of Southampton, Highfield, Southampton SO17 1BJ, United Kingdom*

E-mail: tjvbu@kemi.dtu.dk; kjellgren@sdu.dk; K.M.Ziems@soton.ac.uk

January 12, 2026

Contents

S1 Additional tables and figures **S3**

S1.1 H_2 ladders S3

S1.2 Other molecules S6

S1.3 Strongly correlated systems S10

S1.4 Shot noise S22

S1 Additional tables and figures

S1.1 H_2 ladders

Table S1: Mean absolute errors (MAE) and standard deviations (σ) for the eight RDM and RDC approximations for a maximum of 100 non-zero excitation energies and their corresponding oscillator strengths. Errors and standard deviations for excitation energies are given in eV. The number of zero excitation energies for the molecules and approximations are also given.

		RDM approximations				RDC approximations			
System	Errors	4-z	3- & 4-z	4-d	3- & 4-d	4-z	3- & 4-z	4-d	3- & 4-d
	MAE_{exc}	0.0	0.0	0.0	0.0	0.0	0.0	0.0	0.0
	σ_{exc}	0.0	0.0	0.0	0.0	0.0	0.0	0.0	0.0
1 H_2	MAE_{osc}	0.0	0.0	0.0	0.0	0.0	0.0	0.0	0.0
	σ_{osc}	0.0	0.0	0.0	0.0	0.0	0.0	0.0	0.0
	# zero	0	0	0	0	0	0	0	0
	MAE_{exc}	0.2999	4.2836	0.2999	2.2334	0.2999	4.2835	0.2999	2.2291
	σ_{exc}	0.4434	5.9992	0.4434	3.1977	0.4434	5.9992	0.4434	3.1797
2 H_2	MAE_{osc}	0.0001	0.1901	0.0001	0.1893	0.0001	0.1901	0.0001	0.1893
	σ_{osc}	0.0005	0.4481	0.0005	0.4934	0.0005	0.4481	0.0005	0.4934
	# zero	0	0	0	0	0	0	0	0
	MAE_{exc}	0.4273	7.4679	0.4273	1.6097	0.4414	7.0933	0.4296	1.7333
	σ_{exc}	0.5337	8.4484	0.5337	2.2472	0.5422	8.1492	0.5349	2.3449
3 H_2	MAE_{osc}	0.0002	0.096	0.0002	0.0855	0.0002	0.0941	0.0002	0.0849
	σ_{osc}	0.0005	0.3731	0.0005	0.4096	0.0005	0.3816	0.0005	0.4209
	# zero	0	0	0	0	0	0	0	0
	MAE_{exc}	0.8669	9.6414	0.8669	4.9432	0.8443	8.2002	0.8443	2.0725
	σ_{exc}	1.1659	9.9665	1.1659	5.3266	1.1566	8.4840	1.1566	2.3465
4 H_2	MAE_{osc}	0.0088	0.1303	0.0088	0.1307	0.0087	0.1217	0.0087	0.1741
	σ_{osc}	0.0350	0.3568	0.0350	0.3165	0.0350	0.3268	0.0350	0.5415
	# zero	0	5	0	5	0	5	0	7
	MAE_{exc}	0.6302	12.4120	0.6302	4.8132	0.6972	10.4634	0.6972	2.7017
	σ_{exc}	0.6825	12.4368	0.6825	4.8742	0.7537	10.4870	0.7537	2.8664
5 H_2	MAE_{osc}	0.0017	0.0556	0.0017	0.0884	0.0020	0.0721	0.0020	0.0959
	σ_{osc}	0.0050	0.3924	0.0050	0.4355	0.0053	0.4025	0.0053	0.5406
	# zero	0	0	0	0	0	0	0	0
	MAE_{exc}	0.8699	15.5930	0.8699	8.0726	0.8781	14.0385	0.8781	3.1770
	σ_{exc}	0.9300	15.726	0.9300	8.1374	0.9386	14.1251	0.9386	3.2739
6 H_2	MAE_{osc}	0.1177	0.0709	0.1177	0.0971	0.1177	0.0762	0.1177	0.0883
	σ_{osc}	0.6426	0.4572	0.6426	0.4627	0.6426	0.4425	0.6426	0.4696
	# zero	0	3	0	0	0	0	0	0

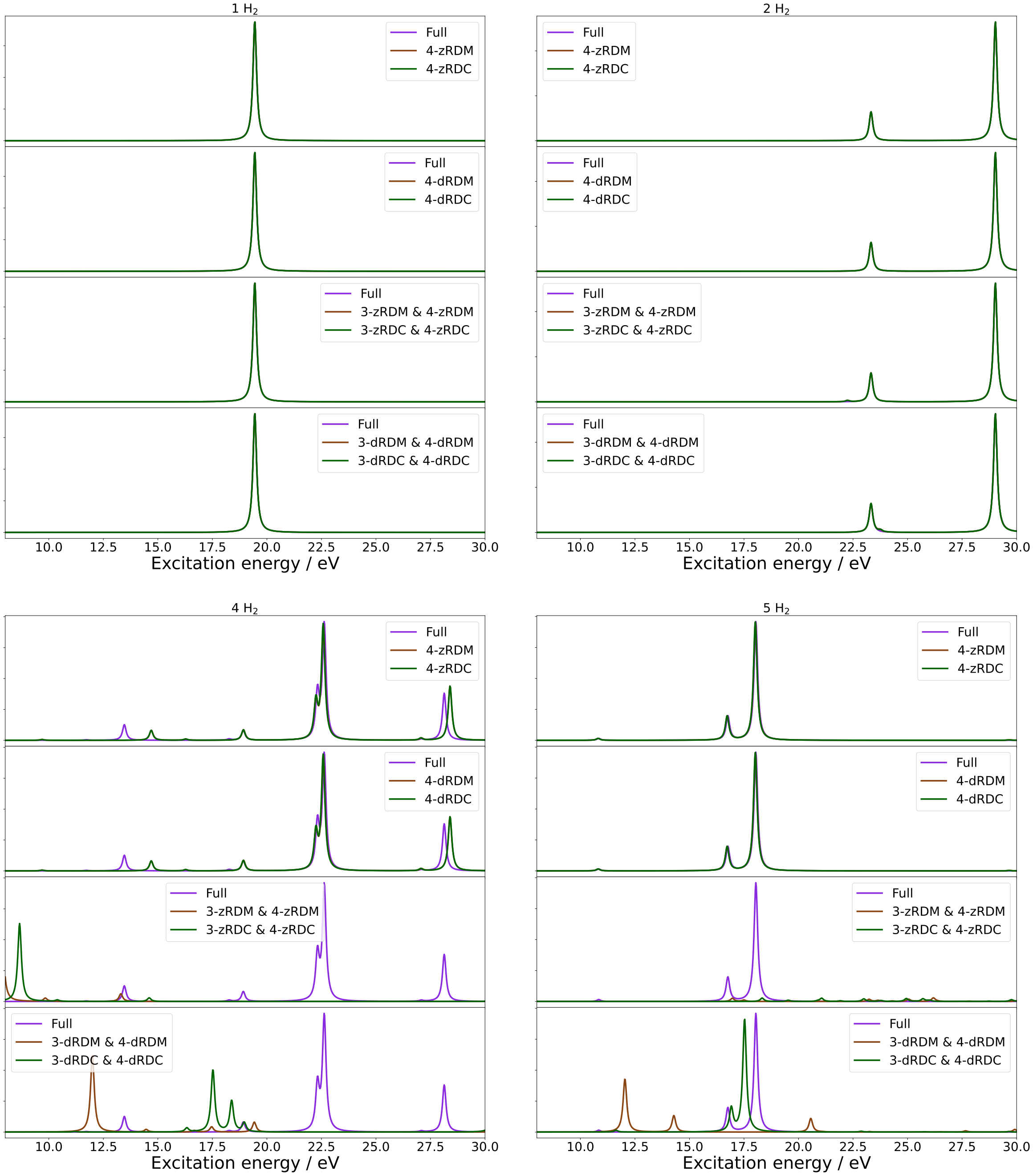

Figure S1: Absorption spectra of H_2 ladders containing one link (top left), two links (top right), four links (bottom left), and five links (bottom right) using the naive qLRSD method on top of a FCI wave function. The four panels in each subfigure contain: (first panel) the absorption spectrum of naive qLRSD using the 4-zRDM and 4-zRDC approximations, (second panel) the absorption spectrum of naive qLRSD using the 4-dRDM and 4-dRDC approximations, (third panel) absorption spectrum of naive qLRSD using the 3-zRDM & 4-zRDM and 3-zRDC & 4-zRDC approximations, and (fourth panel) absorption spectrum of naive qLRSD using the 3-dRDM & 4-dRDM and 3-dRDC & 4-dRDC approximations.

Table S2: Mean absolute errors (MAE) in eV for the eight RDM and RDC approximations for all bright states (osc. str. > 0.01) below 30 eV for different lengths of the H_2 ladder.

	RDM approximations				RDC approximations			
System	4-z	3- & 4-z	4-d	3- & 4-d	4-z	3- & 4-z	4-d	3- & 4-d
1 H_2	0.0000	0.0000	0.0000	0.0000	0.0000	0.0000	0.0000	0.0000
2 H_2	0.0000	0.0000	0.0000	0.0000	0.0000	0.0000	0.0000	0.0000
3 H_2	0.0200	2.8496	0.0200	0.6934	0.0202	2.2036	0.0201	0.5931
4 H_2	0.3241	12.9128	0.3241	0.9846	0.3254	14.5711	0.3254	2.6516
5 H_2	0.0253	16.2148	0.0253	4.2404	0.0298	12.8308	0.0298	0.3383
6 H_2	0.0437	0.0858	0.0437	5.4615	0.0458	9.7298	0.0458	1.7760

Table S3: Minimum and maximum eigenvalues of the Hessian and metric of 4 H_2 at all levels of approximations.

Approximation	Hessian [min, max] eigenvalue	Metric [min, max] eigenvalue
Full	$[5.900 \cdot 10^{-3}, 4.507]$	$[-1.035, 1.035]$
4-zRDM	$[3.753 \cdot 10^{-3}, 4.486]$	$[-1.035, 1.035]$
4-zRDC	$[3.846 \cdot 10^{-3}, 4.486]$	$[-1.035, 1.035]$
3- & 4-zRDM	$[-0.1634, 3.929]$	$[-1.042, 1.042]$
3- & 4-zRDC	$[-0.1638, 3.929]$	$[-1.036, 1.036]$
4-dRDM	$[3.753 \cdot 10^{-3}, 4.486]$	$[-1.035, 1.035]$
4-dRDC	$[3.846 \cdot 10^{-3}, 4.486]$	$[-1.035, 1.035]$
3- & 4-dRDM	$[-0.1318, 4.270]$	$[-1.042, 1.042]$
3- & 4-dRDC	$[-0.1348, 4.172]$	$[-1.036, 1.036]$

S1.2 Other molecules

Table S4: Mean absolute errors (MAE) and standard deviations (σ) for the eight RDM and RDC approximations for a maximum of 100 non-zero excitation energies and their corresponding oscillator strengths. Errors and standard deviations for excitation energies are given in eV. The number of zero excitation energies for the molecules and approximations are also given.

		RDM approximations				RDC approximations			
System	Errors	4-z	3- & 4-z	4-d	3- & 4-d	4-z	3- & 4-z	4-d	3- & 4-d
H_2S (4, 4)	MAE_{exc}	0.0748	1.0614	0.0748	0.6222	0.0748	1.0614	0.0748	0.6227
	σ_{exc}	0.2095	2.4048	0.2095	1.2987	0.2095	2.4048	0.2095	1.300
	MAE_{osc}	0.0008	0.0416	0.0008	0.0255	0.0008	0.0416	0.0008	0.0255
	σ_{osc}	0.0024	0.1001	0.0024	0.0601	0.0024	0.1001	0.0024	0.0601
	# zero	0	0	0	0	0	0	0	0
H_2S (8, 6)	MAE_{exc}	0.3286	1.4537	0.3286	1.1384	0.3295	1.3105	0.3295	1.1497
	σ_{exc}	0.5281	3.2104	0.5281	1.8241	0.5277	2.8022	0.5277	1.9103
	MAE_{osc}	0.0079	0.0677	0.0079	0.0377	0.0079	0.0619	0.0079	0.0087
	σ_{osc}	0.0341	0.1987	0.0341	0.1046	0.0341	0.1919	0.0341	0.0231
	# zero	0	0	0	0	0	0	0	0
OCS (4, 4)	MAE_{exc}	0.0835	26.5346	0.0835	25.3310	0.0835	26.5340	0.0835	25.3311
	σ_{exc}	0.2729	191.3843	0.2729	191.3641	0.2729	191.3843	0.2729	191.3641
	MAE_{osc}	0.0540	0.0808	0.0540	0.0936	0.0540	0.0808	0.0540	0.0935
	σ_{osc}	0.3168	0.2799	0.3168	0.3387	0.3168	0.2800	0.3168	0.3373
	# zero	0	1	0	1	0	1	0	1

OCS (6, 6)	MAE_{exc}	0.5964	10.1088	0.5964	5.9769	0.5934	13.3942	0.5934	3.8050
	σ_{exc}	0.8670	11.9536	0.8670	13.7280	0.8638	20.6362	0.8638	9.2573
	MAE_{osc}	0.0232	0.1065	0.0232	0.0951	0.0231	0.1088	0.0231	0.0934
	σ_{osc}	0.0754	0.3689	0.0754	0.2585	0.0752	0.3883	0.0752	0.3147
	# zero	0	0	0	2	0	3	0	1
SeH_2 (4, 4)	MAE_{exc}	0.0368	0.4996	0.0368	0.3318	0.0368	0.4995	0.0368	0.3318
	σ_{exc}	0.1426	1.5483	0.1426	0.8333	0.1426	1.5483	0.1426	0.8335
	MAE_{osc}	0.0010	0.0235	0.0010	0.0193	0.0010	0.0235	0.0010	0.0193
	σ_{osc}	0.0047	0.0740	0.0047	0.0576	0.0047	0.0741	0.0047	0.0576
	# zero	0	0	0	0	0	0	0	0
SeH_2 (8, 6)	MAE_{exc}	0.2327	1.1153	0.2327	0.6882	0.2341	1.0362	0.2341	0.8265
	σ_{exc}	0.4294	1.9450	0.4294	1.2680	0.4304	1.7966	0.4304	1.4642
	MAE_{osc}	0.0068	0.0467	0.0068	0.0231	0.0068	0.0452	0.0068	0.0085
	σ_{osc}	0.0321	0.1616	0.0321	0.0613	0.0321	0.1619	0.0321	0.0229
	# zero	0	0	0	0	0	0	0	0

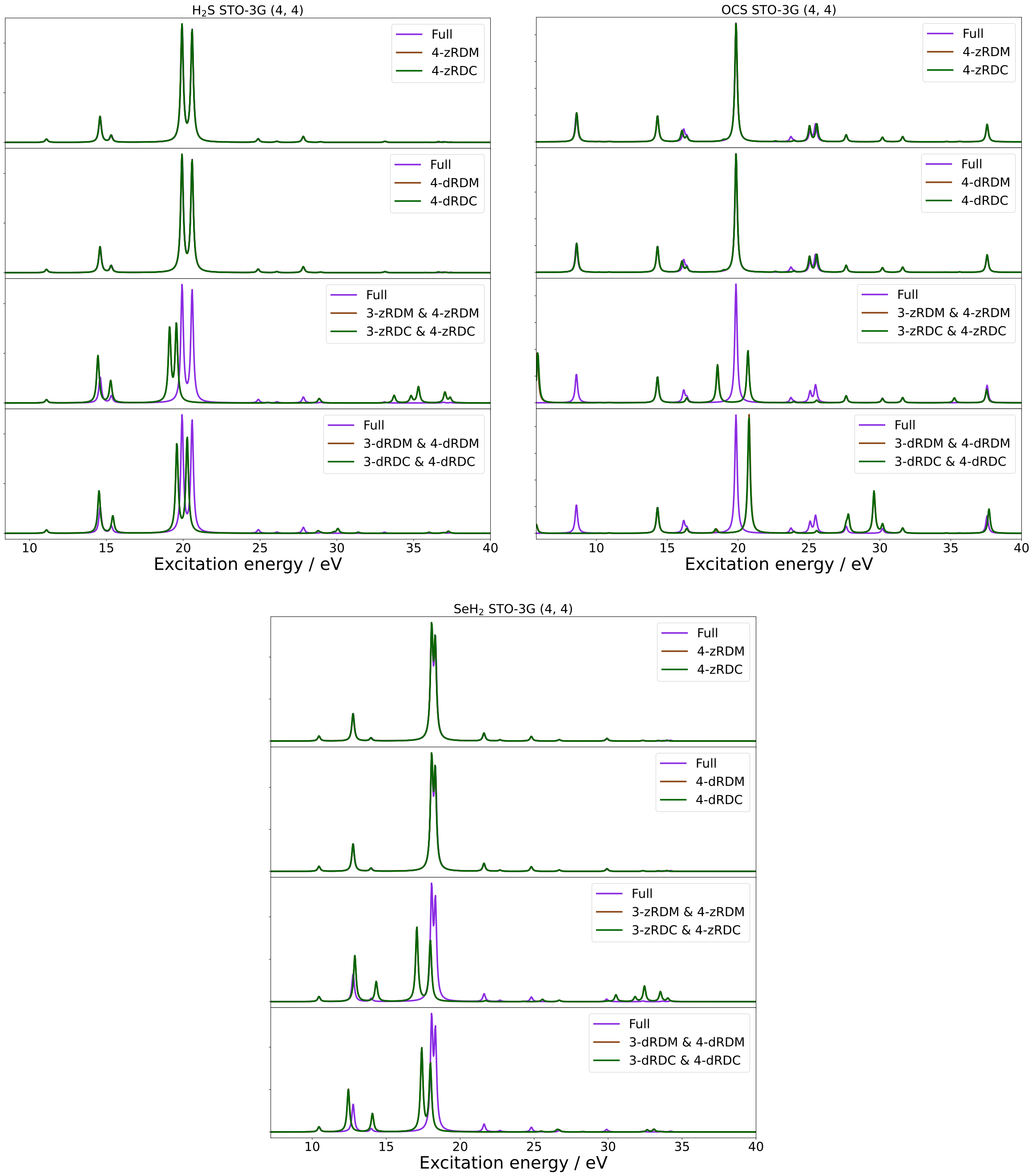

Figure S2: Absorption spectra of H_2S (4, 4) (top left), OCS (4, 4) (top right), and SeH_2 (4, 4) (bottom) using the naive qLRSD method on top of a FCI wave function. The four panels contain: (first panel) the absorption spectrum of naive qLRSD using the 4-zRDM and 4-zRDC approximations; (second panel) the absorption spectrum of naive qLRSD using the 4-dRDM and 4-dRDC approximations; (third panel) absorption spectrum of naive qLRSD using the 3-zRDM & 4-zRDM and 3-zRDC & 4-zRDC approximations; (fourth panel) absorption spectrum of naive qLRSD using the 3-dRDM & 4-dRDM and 3-dRDC & 4-dRDC approximations.

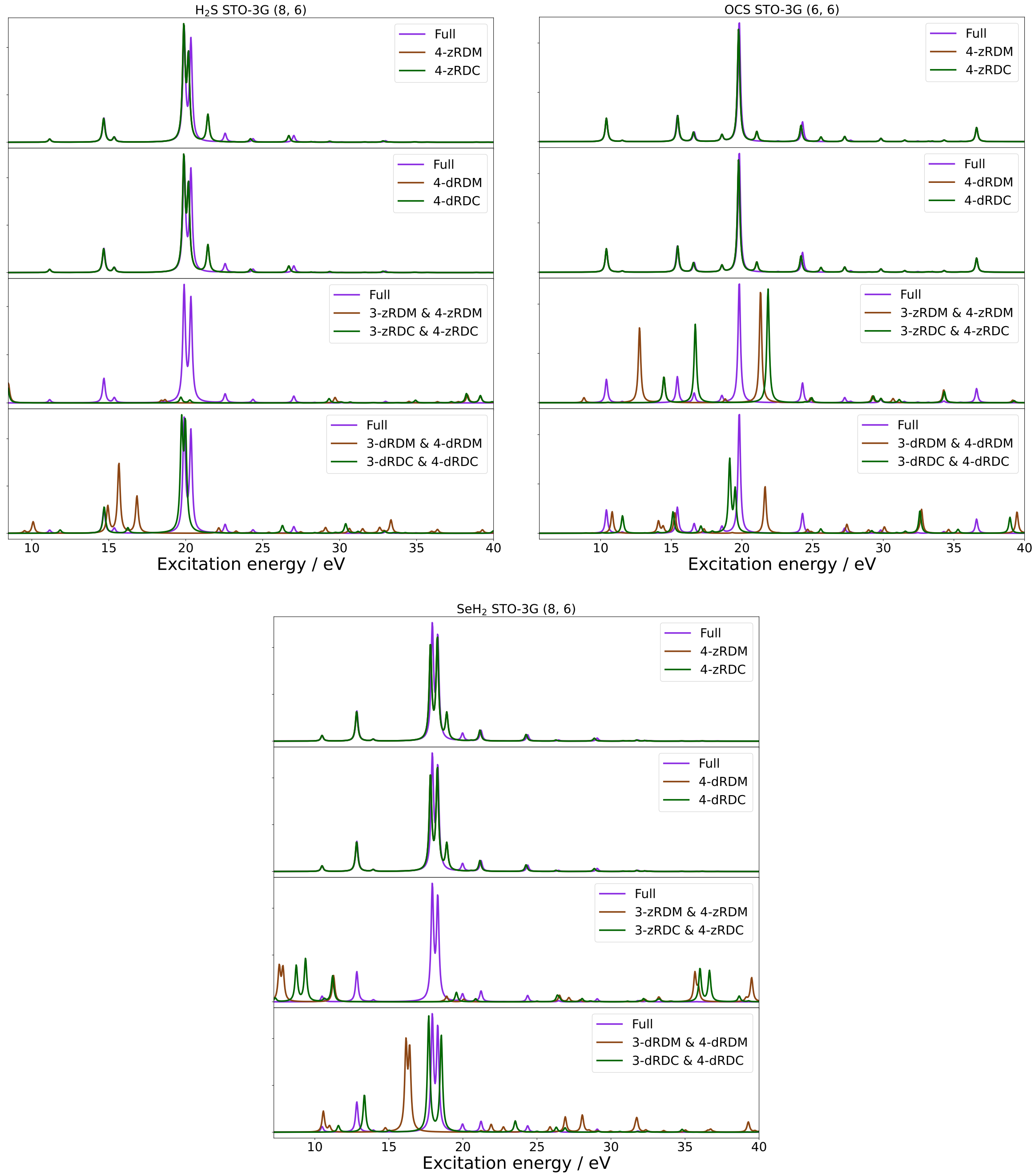

Figure S3: Absorption spectrum of H_2S (8, 6) (top left), OCS (6, 6) (top right), and SeH_2 (bottom). The four panels contain: (first panel) the absorption spectrum of naive qLRSD using the 4-zRDM and 4-zRDC approximations; (second panel) the absorption spectrum of naive qLRSD using the 4-dRDM and 4-dRDC approximations; (third panel) absorption spectrum of naive qLRSD using the 3-zRDM & 4-zRDM and 3-zRDC & 4-zRDC approximations; (fourth panel) absorption spectrum of naive qLRSD using the 3-dRDM & 4-dRDM and 3-dRDC & 4-dRDC approximations.

S1.3 Strongly correlated systems

Table S5: Mean absolute errors (MAE) and standard deviations (σ) for the eight RDM and RDC approximations for the valence excitation energies of H_2O between 5 and 16 eV and their corresponding oscillator strengths. Errors and standard deviations for excitation energies are given in eV.

		RDM approximations				RDC approximations			
System	Errors	4-z	3- & 4-z	4-d	3- & 4-d	4-z	3- & 4-z	4-d	3- & 4-d
H_2O	MAE_{exc}	0.0039	9.3353	0.0039	3.9895	0.0039	9.7281	0.0039	1.1694
1.0 R_{eq}	σ_{exc}	0.0047	9.3709	0.0047	4.1137	0.0047	9.8976	0.0047	1.2396
(6, 6)	MAE_{osc}	0.0001	0.1095	0.0001	0.0339	0.0001	0.1160	0.0001	0.0111
cc-pVDZ	σ_{osc}	0.0002	0.1509	0.0002	0.0532	0.0002	0.1535	0.0002	0.0187
H_2O	MAE_{exc}	0.8934	3.8309	0.8934	5.9632	0.8892	2.7746	0.8892	4.4980
1.5 R_{eq}	σ_{exc}	1.3366	4.1510	1.3366	6.1138	1.3314	3.2528	1.3314	4.8028
(6, 6)	MAE_{osc}	0.0290	0.1304	0.0290	0.1435	0.0290	0.1357	0.0290	0.1227
cc-pVDZ	σ_{osc}	0.0360	0.2749	0.0360	0.2861	0.0360	0.2697	0.0360	0.2148
H_2O	MAE_{exc}	4.6683	6.8834	4.6683	7.0572	4.6598	6.4709	4.6598	7.5589
2.0 R_{eq}	σ_{exc}	4.7913	7.0073	4.7913	7.1314	4.7837	6.5909	4.7837	7.8070
(6, 6)	MAE_{osc}	0.0988	0.0872	0.0988	1.4804	0.0988	0.0786	0.0988	0.0592
cc-pVDZ	σ_{osc}	0.2014	0.1715	0.2014	4.0236	0.2014	0.1689	0.2014	0.1577

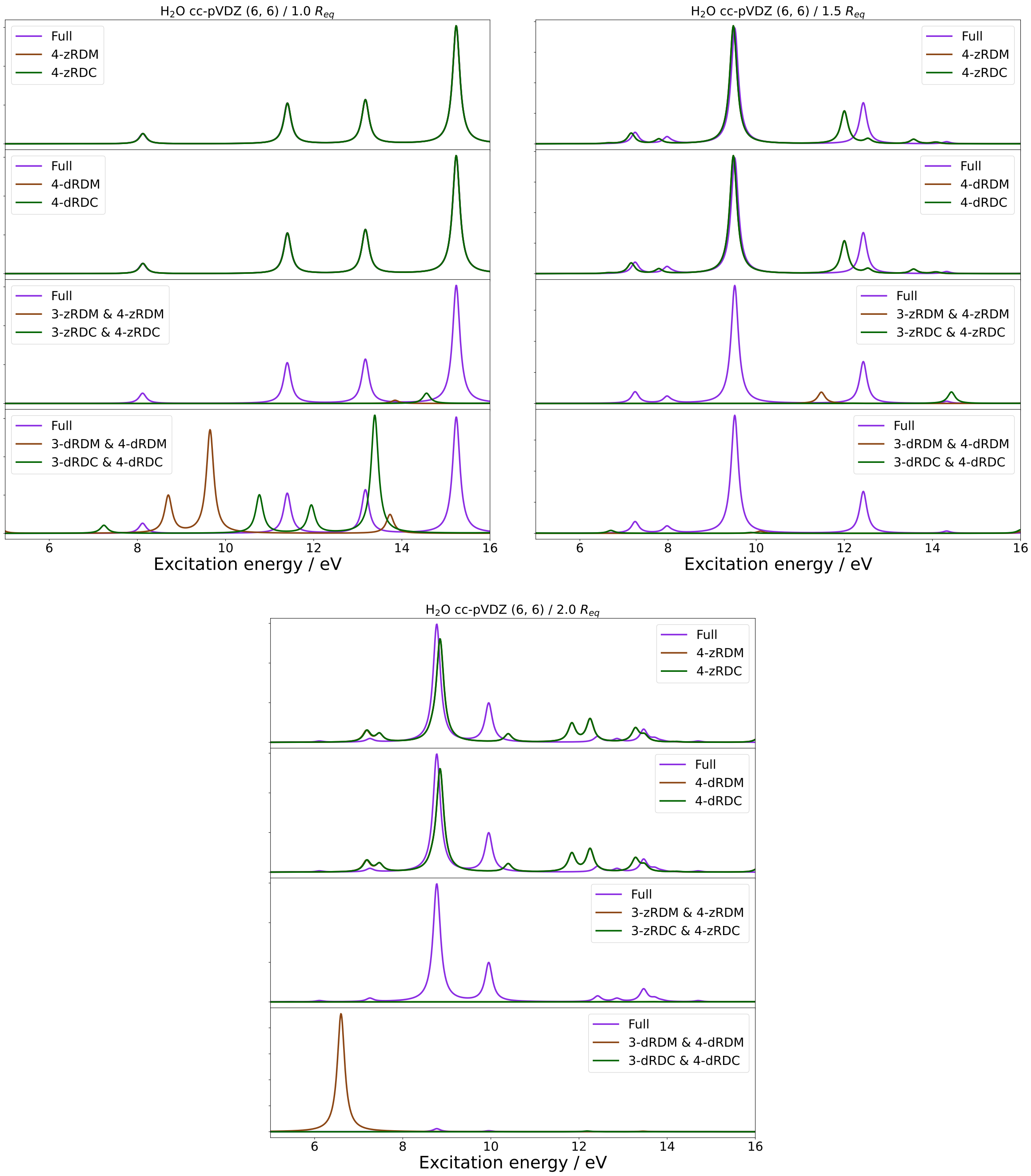

Figure S4: Absorption spectra in the valence excitation region of H_2O in a (6, 6) active space with the cc-pVDZ basis set at differing symmetric O-H stretches. Each figure contains four panels comparing the naive qLRSD absorption spectrum with no approximation to the absorption spectrum of naive qLRSD using the 4-zRDM and 4-zRDC approximations (first panel), the absorption spectrum of naive qLRSD using the 4-dRDM and 4-dRDC approximations (second panel), absorption spectrum of naive qLRSD using the 3-zRDM & 4-zRDM and 3-zRDC & 4-zRDC approximations, (third panel) and absorption spectrum of naive qLRSD using the 3-dRDM & 4-dRDM and 3-dRDC & 4-dRDC approximations (fourth panel).

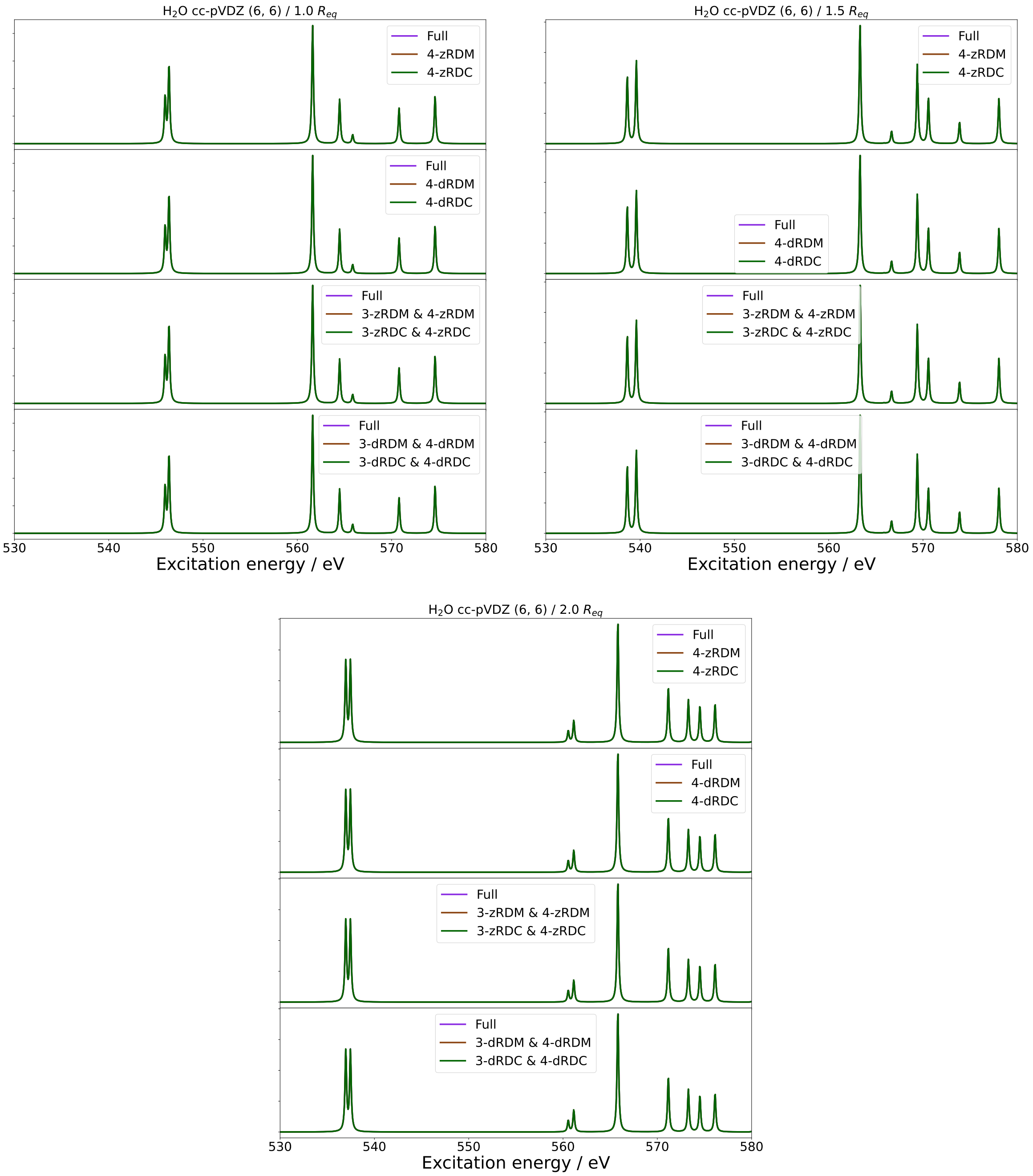

Figure S5: Oxygen K-edge absorption spectra of H_2O in a (6, 6) active space with the cc-pVDZ basis set at differing symmetric O-H stretches. Each figure contains four panels comparing the naive qLRSD absorption spectrum with no approximation to the absorption spectrum of naive qLRSD using the 4-zRDM and 4-zRDC approximations (first panel), the absorption spectrum of naive qLRSD using the 4-dRDM and 4-dRDC approximations (second panel), absorption spectrum of naive qLRSD using the 3-zRDM & 4-zRDM and 3-zRDC & 4-zRDC approximations, (third panel) and absorption spectrum of naive qLRSD using the 3-dRDM & 4-dRDM and 3-dRDC & 4-dRDC approximations (fourth panel).

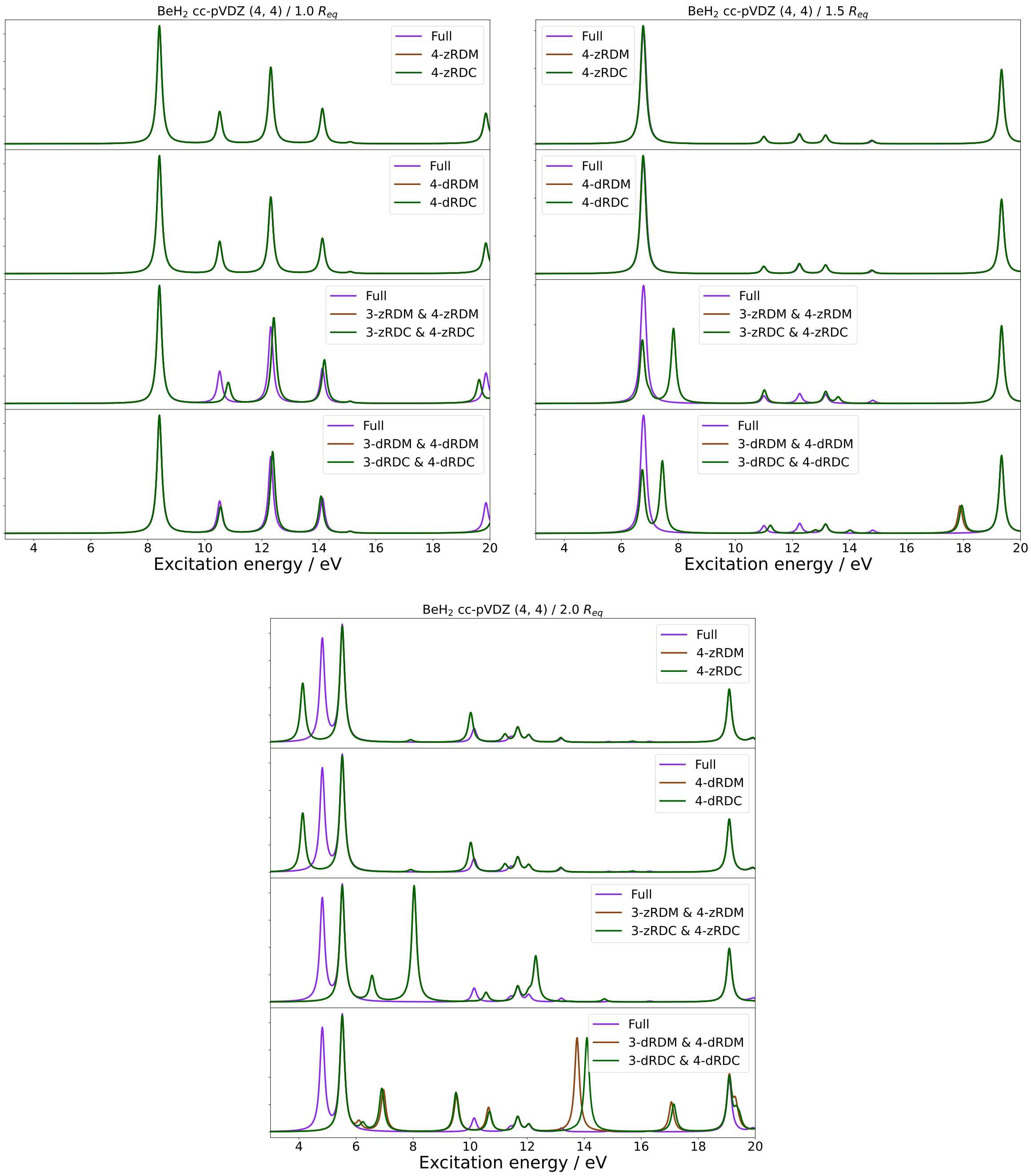

Figure S6: Absorption spectra in the valence excitation region of BeH_2 in a (4, 4) active space with the cc-pVDZ basis set at differing symmetric Be-H stretches. Each figure contains four panels comparing the naive qLRSD absorption spectrum with no approximation to the absorption spectrum of naive qLRSD using the 4-zRDM and 4-zRDC approximations (first panel), the absorption spectrum of naive qLRSD using the 4-dRDM and 4-dRDC approximations (second panel), absorption spectrum of naive qLRSD using the 3-zRDM & 4-zRDM and 3-zRDC & 4-zRDC approximations, (third panel) and absorption spectrum of naive qLRSD using the 3-dRDM & 4-dRDM and 3-dRDC & 4-dRDC approximations (fourth panel).

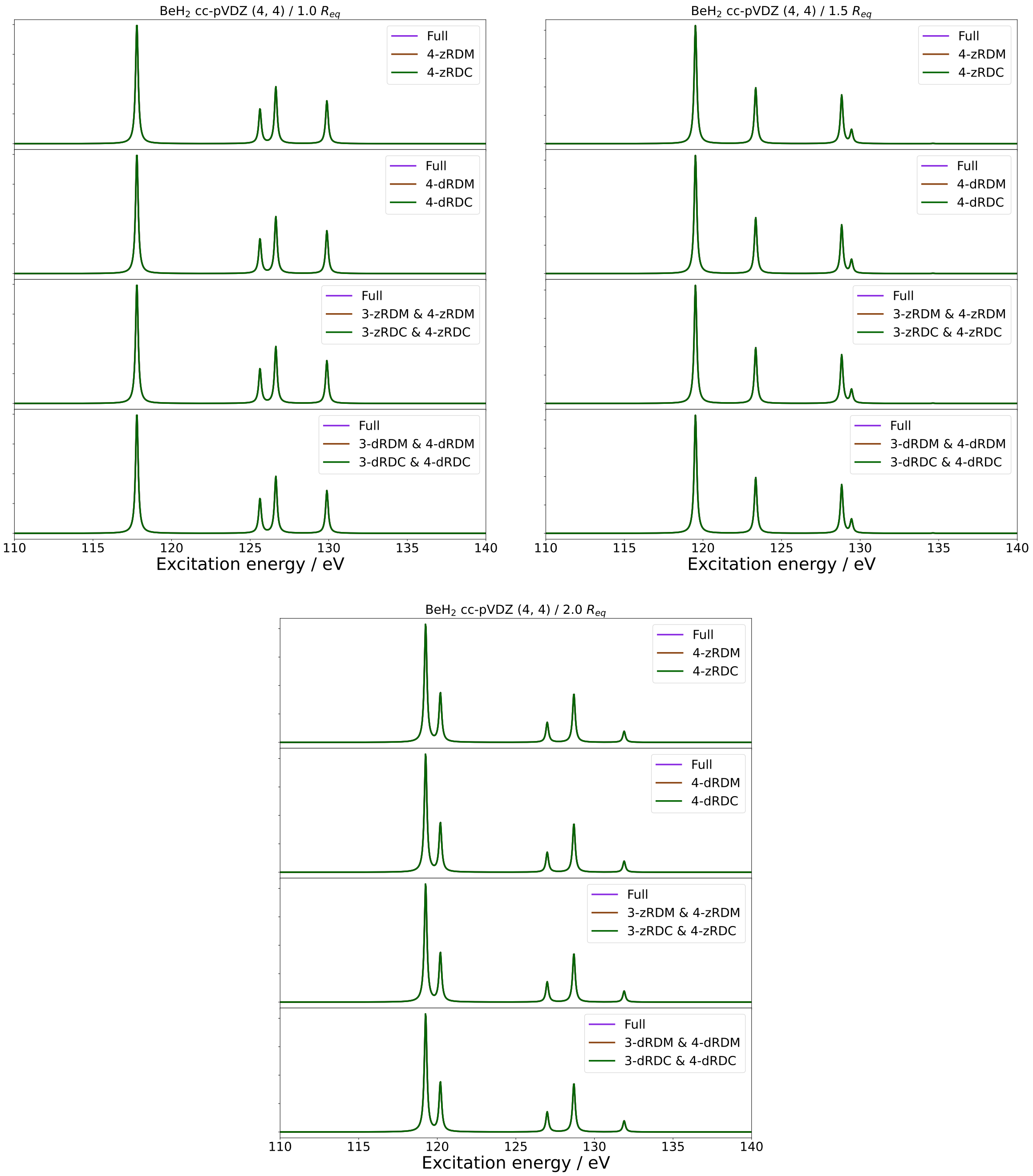

Figure S7: Beryllium K-edge absorption spectra of BeH_2 in a (4, 4) active space with the cc-pVDZ basis set at differing symmetric Be-H stretches. Each figure contains four panels comparing the naive qLRSD absorption spectrum with no approximation to the absorption spectrum of naive qLRSD using the 4-zRDM and 4-zRDC approximations (first panel), the absorption spectrum of naive qLRSD using the 4-dRDM and 4-dRDC approximations (second panel), absorption spectrum of naive qLRSD using the 3-zRDM & 4-zRDM and 3-zRDC & 4-zRDC approximations, (third panel) and absorption spectrum of naive qLRSD using the 3-dRDM & 4-dRDM and 3-dRDC & 4-dRDC approximations (fourth panel).

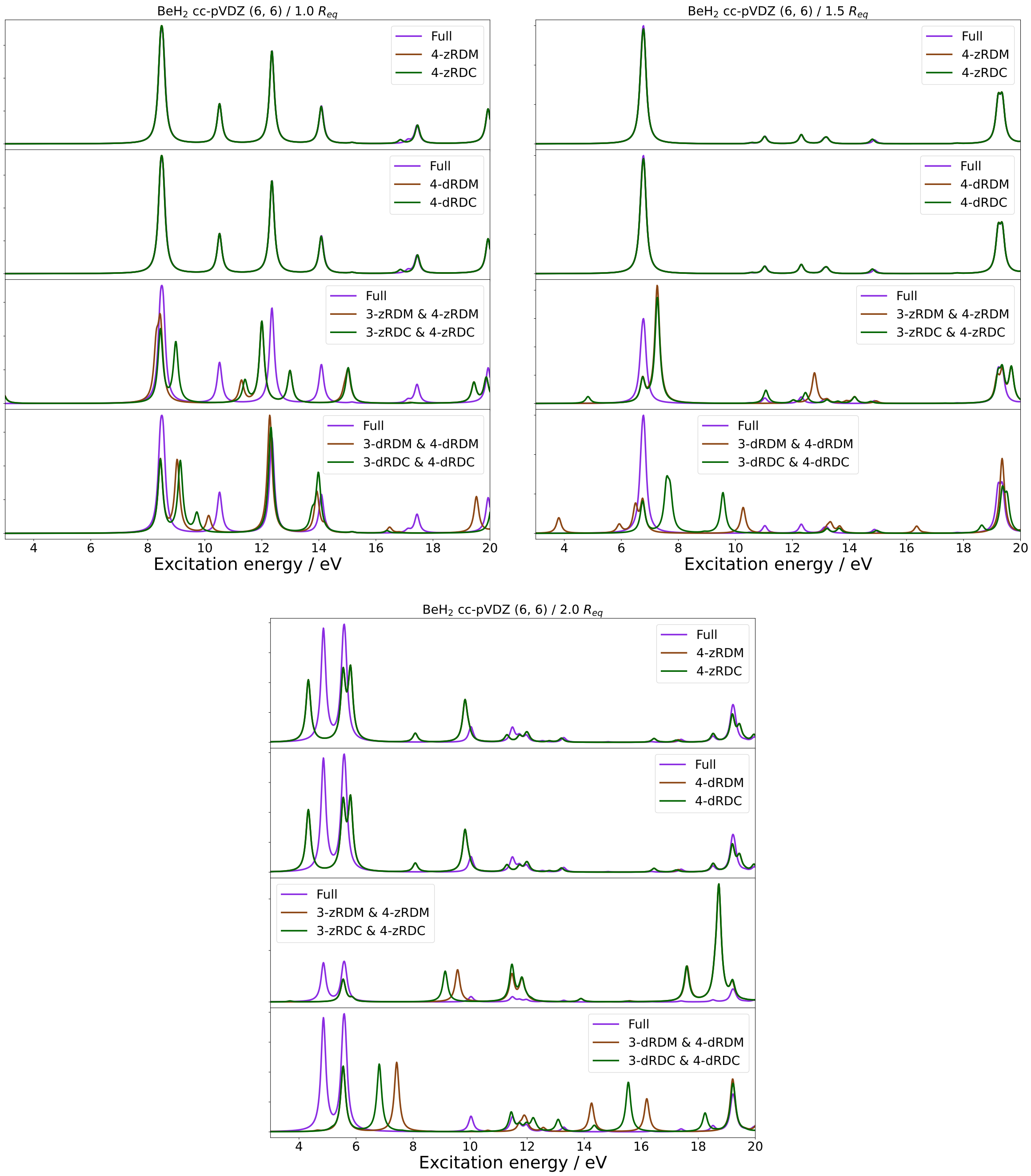

Figure S8: Absorption spectra in the valence excitation region of BeH_2 in a (6, 6) active space with the cc-pVDZ basis set at differing symmetric Be-H stretches. Each figure contains four panels comparing the naive qLRSD absorption spectrum with no approximation to the absorption spectrum of naive qLRSD using the 4-zRDM and 4-zRDC approximations (first panel), the absorption spectrum of naive qLRSD using the 4-dRDM and 4-dRDC approximations (second panel), absorption spectrum of naive qLRSD using the 3-zRDM & 4-zRDM and 3-zRDC & 4-zRDC approximations, (third panel) and absorption spectrum of naive qLRSD using the 3-dRDM & 4-dRDM and 3-dRDC & 4-dRDC approximations (fourth panel).

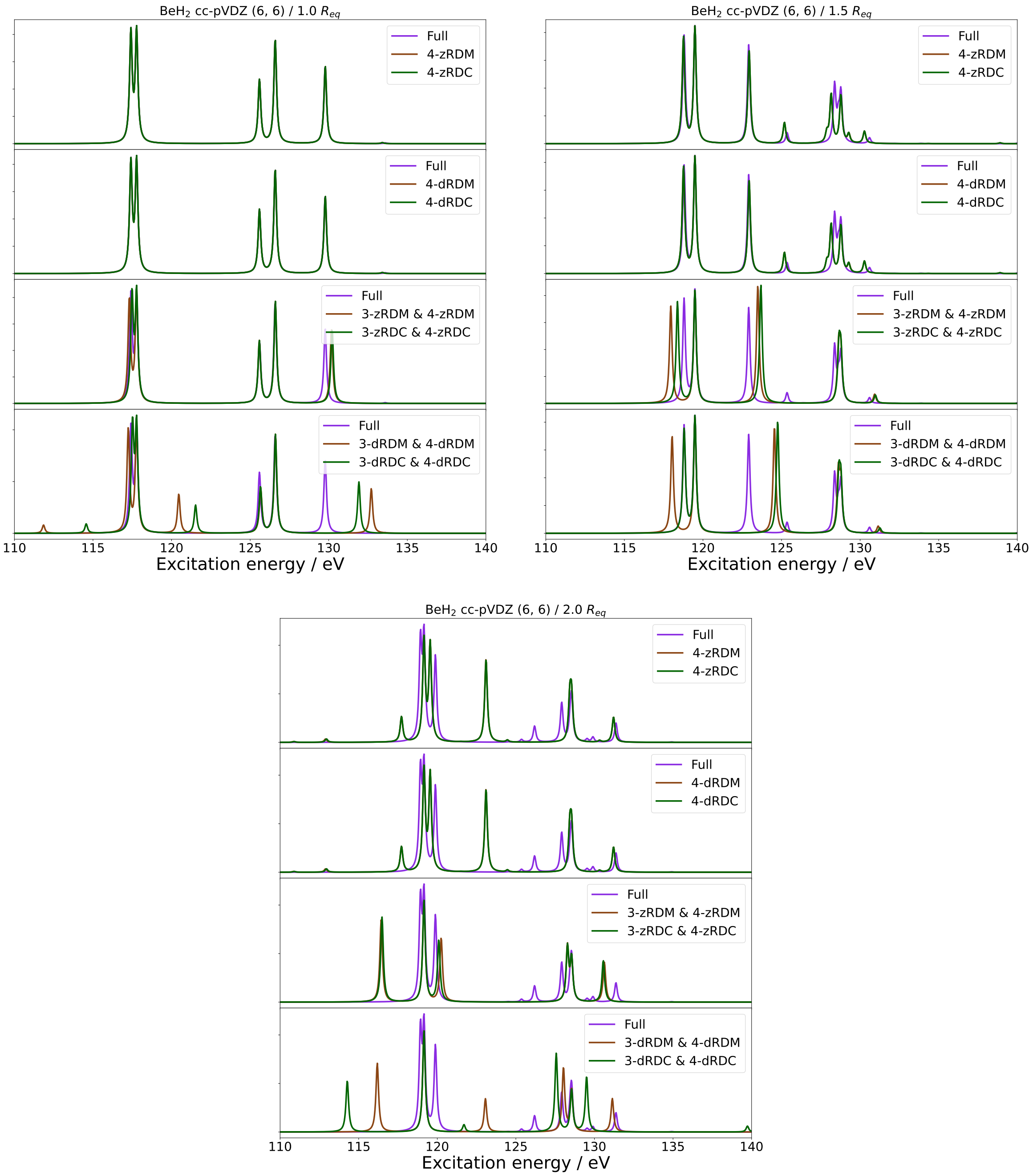

Figure S9: Beryllium K-edge absorption spectra of BeH_2 in a (6, 6) active space with the cc-pVDZ basis set at differing symmetric Be-H stretches. Each figure contains four panels comparing the naive qLRSD absorption spectrum with no approximation to the absorption spectrum of naive qLRSD using the 4-zRDM and 4-zRDC approximations (first panel), the absorption spectrum of naive qLRSD using the 4-dRDM and 4-dRDC approximations (second panel), absorption spectrum of naive qLRSD using the 3-zRDM & 4-zRDM and 3-zRDC & 4-zRDC approximations, (third panel) and absorption spectrum of naive qLRSD using the 3-dRDM & 4-dRDM and 3-dRDC & 4-dRDC approximations (fourth panel).

Table S6: Mean absolute errors (MAE) and standard deviations (σ) for the eight RDM and RDC approximations for the oxygen K-edge excitation energies of H_2O between 530 and 600 eV and their corresponding oscillator strengths. Errors and standard deviations for excitation energies are given in eV.

		RDM approximations				RDC approximations			
System	Errors	4-z	3- & 4-z	4-d	3- & 4-d	4-z	3- & 4-z	4-d	3- & 4-d
H_2O	MAE_{exc}	0.0	0.0002	0.0	0.0003	0.0	0.0002	0.0	0.0002
1.0 R_{eq}	σ_{exc}	0.0	0.0003	0.0	0.0005	0.0	0.0002	0.0	0.0003
(6, 6)	MAE_{osc}	0.0	0.0001	0.0	0.0001	0.0	0.0001	0.0	0.0001
cc-pVDZ	σ_{osc}	0.0	0.0001	0.0	0.0002	0.0	0.0001	0.0	0.0001
H_2O	MAE_{exc}	0.0	0.0001	0.0	0.0002	0.0	0.0001	0.0	0.0001
1.5 R_{eq}	σ_{exc}	0.0	0.0002	0.0	0.0002	0.0	0.0002	0.0	0.0002
(6, 6)	MAE_{osc}	0.0	0.0001	0.0	0.0001	0.0	0.0001	0.0	0.0001
cc-pVDZ	σ_{osc}	0.0	0.0002	0.0	0.0001	0.0	0.0001	0.0	0.0001
H_2O	MAE_{exc}	0.0	0.0001	0.0	0.0001	0.0	0.0001	0.0	0.0001
2.0 R_{eq}	σ_{exc}	0.0	0.0002	0.0	0.0002	0.0	0.0002	0.0	0.0002
(6, 6)	MAE_{osc}	0.0	0.0001	0.0	0.0001	0.0	0.0001	0.0	0.0001
cc-pVDZ	σ_{osc}	0.0	0.0001	0.0	0.0001	0.0	0.0001	0.0	0.0001

Table S7: Mean absolute errors (MAE) and standard deviations (σ) for the eight RDM and RDC approximations for the valence excitation energies of BeH_2 between 3 and 20 eV and their corresponding oscillator strengths. Errors and standard deviations for excitation energies are given in eV.

		RDM approximations				RDC approximations			
System	Errors	4-z	3- & 4-z	4-d	3- & 4-d	4-z	3- & 4-z	4-d	3- & 4-d
BeH_2	MAE_{exc}	0.0010	0.0929	0.0010	0.0532	0.0010	0.0929	0.0010	0.0529
1.0 R_{eq}	σ_{exc}	0.0019	0.1561	0.0019	0.0962	0.0019	0.1561	0.0019	0.0958
(4, 4)	MAE_{osc}	0.0001	0.0157	0.0001	0.0074	0.0001	0.0157	0.0001	0.0074
cc-pVDZ	σ_{osc}	0.0003	0.0323	0.0003	0.0158	0.0003	0.0323	0.0003	0.0158
BeH_2	MAE_{exc}	0.0191	0.7957	0.0191	0.3714	0.0191	0.7955	0.0191	0.3733
1.5 R_{eq}	σ_{exc}	0.0514	1.2285	0.0514	0.7086	0.0514	1.2280	0.0514	0.7117
(4, 4)	MAE_{osc}	0.0042	0.1233	0.0042	0.0576	0.0042	0.1233	0.0042	0.0583
cc-pVDZ	σ_{osc}	0.0124	0.2837	0.0124	0.1521	0.0124	0.2837	0.0124	0.1525
BeH_2	MAE_{exc}	0.1447	1.2517	0.1447	0.4398	0.1447	1.2514	0.1447	0.3965
2.0 R_{eq}	σ_{exc}	0.3397	1.7056	0.3397	0.7071	0.3397	1.7071	0.3397	0.6418
(4, 4)	MAE_{osc}	0.0137	0.0786	0.0137	0.0784	0.0137	0.0799	0.0137	0.0772
cc-pVDZ	σ_{osc}	0.0544	0.1891	0.0544	0.1835	0.0544	0.1893	0.0544	0.1821
BeH_2	MAE_{exc}	0.0185	5.3726	0.0185	4.0771	0.0185	5.2981	0.0185	4.1017
1.0 R_{eq}	σ_{exc}	0.0595	5.5964	0.0595	4.4768	0.0595	5.5304	0.0595	4.5006
(6, 6)	MAE_{osc}	0.0005	0.1919	0.0005	0.1622	0.0005	0.2011	0.0005	0.2011
cc-pVDZ	σ_{osc}	0.0011	0.2647	0.0011	0.2532	0.0011	0.2711	0.0011	0.2891
BeH_2	MAE_{exc}	0.0273	4.3994	0.0273	4.0462	0.0291	4.1086	0.0291	4.1231
1.5 R_{eq}	σ_{exc}	0.0505	4.5044	0.0505	4.3091	0.0528	4.2918	0.0528	4.1997
(6, 6)	MAE_{osc}	0.0016	0.1745	0.0016	0.1223	0.0315	0.1690	0.0315	0.1479
cc-pVDZ	σ_{osc}	0.0041	0.3414	0.0041	0.2216	0.1212	0.3184	0.1212	0.2725

BeH_2	MAE_{exc}	0.1987	2.3513	0.1987	3.1177	0.1935	2.6061	0.1935	3.5421
2.0 R_{eq}	σ_{exc}	0.3235	2.7690	0.3235	3.7750	0.3057	3.0561	0.3057	3.9298
(6, 6)	MAE_{osc}	0.0286	0.1541	0.0286	0.0848	0.0303	0.1563	0.0303	0.0866
cc-pVDZ	σ_{osc}	0.0742	0.3847	0.0742	0.1770	0.0754	0.3838	0.0754	0.1829

Table S8: Mean absolute errors (MAE) and standard deviations (σ) for the eight RDM and RDC approximations for the beryllium K-edge excitation energies of BeH_2 between 110 and 140 eV and their corresponding oscillator strengths. Errors and standard deviations for excitation energies are given in eV.

		RDM approximations				RDC approximations			
System	Errors	4-z	3- & 4-z	4-d	3- & 4-d	4-z	3- & 4-z	4-d	3- & 4-d
BeH_2	MAE_{exc}	0.0	0.0	0.0	0.0	0.0	0.0	0.0	0.0
1.0 R_{eq}	σ_{exc}	0.0	0.0	0.0	0.0001	0.0	0.0	0.0	0.0001
(4, 4)	MAE_{osc}	0.0	0.0	0.0	0.0	0.0	0.0	0.0	0.0
cc-pVDZ	σ_{osc}	0.0	0.0	0.0	0.0	0.0	0.0	0.0	0.0
BeH_2	MAE_{exc}	0.0	0.0003	0.0	0.0002	0.0	0.0003	0.0	0.0002
1.5 R_{eq}	σ_{exc}	0.0	0.0006	0.0	0.0005	0.0	0.0006	0.0	0.0005
(4, 4)	MAE_{osc}	0.0	0.0	0.0	0.0	0.0	0.0	0.0	0.0
cc-pVDZ	σ_{osc}	0.0	0.0001	0.0	0.0001	0.0	0.0001	0.0	0.0001
BeH_2	MAE_{exc}	0.0	0.0002	0.0	0.0002	0.0	0.0002	0.0	0.0002
2.0 R_{eq}	σ_{exc}	0.0	0.0003	0.0	0.0003	0.0	0.0003	0.0	0.0003
(4, 4)	MAE_{osc}	0.0	0.0001	0.0	0.0001	0.0	0.0001	0.0	0.0001
cc-pVDZ	σ_{osc}	0.0	0.0002	0.0	0.0002	0.0	0.0002	0.0	0.0002
BeH_2	MAE_{exc}	0.0542	4.8742	0.0542	22.1473	0.0539	4.8856	0.0539	20.5622
1.0 R_{eq}	σ_{exc}	0.1250	9.9286	0.1250	23.5241	0.1245	9.9419	0.1245	21.6980
(6, 6)	MAE_{osc}	0.0001	0.0014	0.0001	0.0456	0.0001	0.0013	0.0001	0.0454
cc-pVDZ	σ_{osc}	0.0003	0.0027	0.0003	0.0583	0.0003	0.0026	0.0003	0.0585
BeH_2	MAE_{exc}	0.6947	20.8381	0.6947	19.8295	0.693	20.7460	0.6930	18.8591
1.5 R_{eq}	σ_{exc}	1.0323	26.8827	1.0323	26.5398	1.0326	26.7414	1.0326	25.0223
(6, 6)	MAE_{osc}	0.0051	0.0096	0.0051	0.0304	0.0051	0.0094	0.0051	0.0312
cc-pVDZ	σ_{osc}	0.0131	0.0183	0.0131	0.0481	0.0124	0.0180	0.0124	0.0494

BeH_2	MAE_{exc}	3.3190	22.6587	3.3190	20.1038	3.3008	22.5023	3.3008	18.3609
2.0 R_{eq}	σ_{exc}	4.4316	27.8036	4.4316	27.8887	4.4153	27.5965	4.4153	26.6129
(6, 6)	MAE_{osc}	0.0274	0.0105	0.0274	0.0398	0.0274	0.0106	0.0274	0.0503
cc-pVDZ	σ_{osc}	0.0455	0.0194	0.0455	0.0897	0.0455	0.0195	0.0455	0.1198

S1.4 Shot noise

In this shot noise investigation we will focus on the effects of shot noise on the 3- & 4-zRDM approximation. Here we use the 2 H_2 molecule of the H_2 ladder investigation with a fUCCSD ansatz and the STO-3G basis set and use the Jordan-Wigner mapping. We sample each shot count 50 times. In Fig. S10 we show the effects of an increasing number of shots on no approximations and the 3- & 4-zRDM approximation. In Tables S9 and S10 we show the variance of standard deviation of all excitation energies and their associated oscillator strengths for each shot count. We also provide the total variance and standard deviation across all excitations and oscillator strengths.

As seen in Fig. 1 there is no qualitative error between the spectrum with no approximations and the 3- & 4-zRDM approximated spectrum. In Fig. S10 we see little error between the shot noise spectra and the ideal spectrum for the full RDM simulation (left panel) and the 3- & 4 zRDM approximation (right panel). As expected with an increase in the number of shots (top to bottom panel) the shot noise simulations converge towards the ideal spectrum. In Table S9, we show the variance and standard deviation of the shot noise simulation with no approximation to the RDM algorithm. We see the same trend for each individual excitation energy and oscillator strength, where an increase in the number of shots leads to a decrease in the variance and standard deviation. In Table S10, we show the variance and standard deviation of the shot noise simulation for the 3- & 4-zRDM approximation. We see an increase in errors compared to the non-approximated results. The large error in oscillator strengths of the fourth and fifth excitations are caused by the two states swapping when approximating the 3-RDM. However, it is also clear that for such a small system the error caused by shot noise does not overshadow the error of the RDM approximations themselves.

It is expected that the size of the error caused by shot noise will increase with the size of the active space.

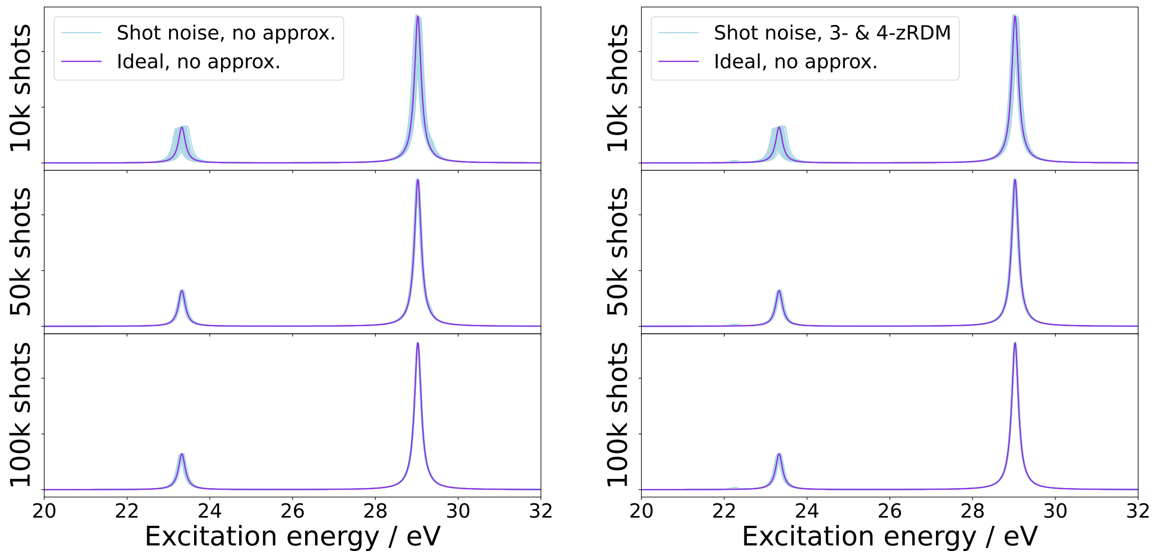

Figure S10: Absorption spectra of 2 H_2 with shot noise using no approximations (left) and the 3- & 4-zRDM approximation (right). The three panels in each subfigure contain: (first panel) 10.000 shots, (second panel) 50.000 shots, and (third panel) 100.000 shots. Each panel contains 50 samples.

Table S9: Ideal excitation energies (Exc, eV) and oscillator strengths (Osc, dimensionless) of 2 H_2 (full spectrum, STO-3G basis) shown in Fig. S10, with the variance and standard deviation of the 50 samples using 10k, 50k, and 100k shots for the excitation energies and oscillator strengths with no approximations.

Ideal		Variance						Standard deviation					
		10k shots		50k shots		100k shots		10k shots		50k shots		100k shots	
Exc.	Osc.	Exc.	Osc.	Exc.	Osc.	Exc.	Osc.	Exc.	Osc.	Exc.	Osc.	Exc.	Osc.
10.1185	0.0000	0.0019	0.0000	0.0002	0.0000	0.0001	0.0000	0.0440	0.0000	0.0156	0.0000	0.0103	0.0000
13.6416	0.0000	0.0012	0.0000	0.0003	0.0000	0.0001	0.0000	0.0348	0.0000	0.0164	0.0000	0.0112	0.0000
23.3246	0.3210	0.0033	0.0000	0.0004	0.0000	0.0003	0.0000	0.0577	0.0063	0.0199	0.0024	0.0180	0.0019
29.0260	1.3184	0.0007	0.0018	0.0001	0.0001	0.0000	0.0000	0.0261	0.0430	0.0115	0.0109	0.0058	0.0067
29.3240	0.0063	0.0036	0.0019	0.0006	0.0001	0.0004	0.0000	0.0598	0.0434	0.0248	0.0100	0.0189	0.0063
35.4102	0.0000	0.0018	0.0000	0.0003	0.0000	0.0001	0.0000	0.0427	0.0004	0.0184	0.0001	0.0122	0.0000
37.0678	0.0297	0.0048	0.0000	0.0008	0.0000	0.0005	0.0000	0.0693	0.0014	0.0282	0.0007	0.0229	0.0005
38.1867	0.0000	0.0017	0.0000	0.0002	0.0000	0.0001	0.0000	0.0408	0.0001	0.0145	0.0000	0.0100	0.0000
45.9914	0.0000	0.0022	0.0000	0.0003	0.0000	0.0002	0.0000	0.0465	0.0001	0.0177	0.0000	0.0134	0.0000
51.6564	0.0000	0.0034	0.0000	0.0008	0.0000	0.0003	0.0000	0.0582	0.0000	0.0280	0.0000	0.0165	0.0000
57.0004	0.0000	0.0079	0.0000	0.0017	0.0000	0.0008	0.0000	0.0892	0.0001	0.0415	0.0000	0.0281	0.0000
58.1965	0.0000	0.0077	0.0000	0.0016	0.0000	0.0009	0.0000	0.0876	0.0001	0.0406	0.0000	0.0304	0.0000
71.7060	0.0001	0.0205	0.0000	0.0050	0.0000	0.0024	0.0000	0.1433	0.0002	0.0705	0.0001	0.0488	0.0000
76.4390	0.0000	0.0087	0.0000	0.0018	0.0000	0.0011	0.0000	0.0932	0.0001	0.0419	0.0000	0.0328	0.0000
Total		0.0050	0.0003	0.0010	0.0000	0.0005	0.0000	0.0704	0.0164	0.0318	0.0040	0.0229	0.0025

Table S10: Ideal excitation energies (Exc, eV) and oscillator strengths (Osc, dimensionless) of 2 H_2 (full spectrum, STO-3G basis) shown in Fig. S10, with the variance and standard deviation of the 50 samples using 10k, 50k, and 100k shots for the excitation energies and oscillator strengths in the 3- & 4-zRDM approximation.

		Variance						Standard deviation					
Ideal		10k shots		50k shots		100k shots		10k shots		50k shots		100k shots	
Exc.	Osc.	Exc.	Osc.	Exc.	Osc.	Exc.	Osc.	Exc.	Osc.	Exc.	Osc.	Exc.	Osc.
10.1185	0.0000	0.0019	0.0000	0.0002	0.0000	0.0001	0.0000	0.0440	0.0000	0.0156	0.0000	0.0103	0.0000
13.6416	0.0000	0.3710	0.0000	0.3733	0.0000	0.3720	0.0000	0.6091	0.0000	0.6110	0.0000	0.6099	0.0000
23.3246	0.3210	1.1628	0.0939	1.1655	0.0940	1.1652	0.0939	1.0783	0.3065	1.0796	0.3066	1.0795	0.3065
29.0260	1.3184	32.5630	0.9952	32.4837	0.9942	32.4695	0.9941	5.7064	0.9976	5.6994	0.9971	5.6982	0.9970
29.3240	0.0063	0.0880	1.7230	0.0883	1.7204	0.0880	1.7206	0.2967	1.3126	0.2971	1.3116	0.2966	1.3117
35.4102	0.0000	3.8257	0.0001	3.8307	0.0001	3.8279	0.0001	1.9559	0.0087	1.9572	0.0085	1.9565	0.0085
37.0678	0.0297	1.2570	0.0009	1.2598	0.0009	1.2569	0.0009	1.1212	0.0297	1.1224	0.0297	1.1211	0.0297
38.1867	0.0000	0.1543	0.0000	0.1536	0.0000	0.1531	0.0000	0.3928	0.0000	0.3920	0.0000	0.3913	0.0000
45.9914	0.0000	26.9751	0.0000	26.9764	0.0000	26.9755	0.0000	5.1938	0.0000	5.1939	0.0000	5.1938	0.0000
51.6564	0.0000	80.7986	0.0000	80.8163	0.0000	80.8212	0.0000	8.9888	0.0001	8.9898	0.0000	8.9901	0.0000
57.0004	0.0000	144.3277	0.0000	144.3508	0.0000	144.3180	0.0000	12.0136	0.0001	12.0146	0.0000	12.0132	0.0000
58.1965	0.0000	15.9957	0.0000	15.9991	0.0000	15.9960	0.0000	3.9995	0.0003	3.9999	0.0002	3.9995	0.0002
71.7060	0.0001	161.3830	0.0000	161.3777	0.0000	161.4007	0.0000	12.7037	0.0070	12.7035	0.0067	12.7044	0.0067
76.4390	0.0000	34.9258	0.0000	34.9174	0.0000	34.9210	0.0000	5.9098	0.0001	5.9091	0.0000	5.9094	0.0000
Total		35.9878	0.2009	35.9852	0.2007	35.9832	0.2007	5.9990	0.4483	5.9988	0.4480	5.9986	0.4480
